# Chloroquine-Inducible Par-4 Secretion Is Essential for Tumor Cell Apoptosis and Inhibition of Metastasis

**DOI:** 10.1016/j.celrep.2016.12.051

**Published:** 2017-01-10

**Authors:** Ravshan Burikhanov, Nikhil Hebbar, Sunil K. Noothi, Nidhi Shukla, James Sledziona, Nathália Araujo, Meghana Kudrimoti, Qing Jun Wang, David S. Watt, Danny R. Welch, Jodi Maranchie, Akihiro Harada, Vivek M. Rangnekar

**Affiliations:** 1 Department of Radiation Medicine, University of Kentucky, Lexington, KY 40356, USA; 2 Graduate Center for Toxicology and Cancer Biology, University of Kentucky, Lexington, KY 40356, USA; 3 Department of Microbiology, Immunology and Molecular Genetics, University of Kentucky, Lexington, KY 40356, USA; 4 Molecular and Cellular Biochemistry, University of Kentucky, Lexington, KY 40356, USA; 5 Lucille Parker Markey Cancer Center, University of Kentucky, Lexington, KY 40356, USA; 6 Department of Cancer Biology, University of Kansas, Kansas City, KS 66160, USA; 7 Department of Urology, University of Pittsburgh, Pittsburgh, PA 15232, USA; 8 Department of Cell Biology, Osaka University, Osaka 565-0871, Japan

## Abstract

The induction of tumor suppressor proteins capable of cancer cell apoptosis represents an attractive option for the re-purposing of existing drugs. We report that the anti-malarial drug, chloroquine (CQ), is a robust inducer of Par-4 secretion from normal cells in mice and cancer patients in a clinical trial. CQ-inducible Par-4 secretion triggers paracrine apoptosis of cancer cells and also inhibits metastatic tumor growth. CQ induces Par-4 secretion via the classical secretory pathway that requires the activation of p53. Mechanistically, p53 directly induces Rab8b, a GTPase essential for vesicle transport of Par-4 to the plasma membrane prior to secretion. Our findings indicate that CQ induces p53- and Rab8b-dependent Par-4 secretion from normal cells for Par-4-dependent inhibition of metastatic tumor growth.

## Graphical abstract

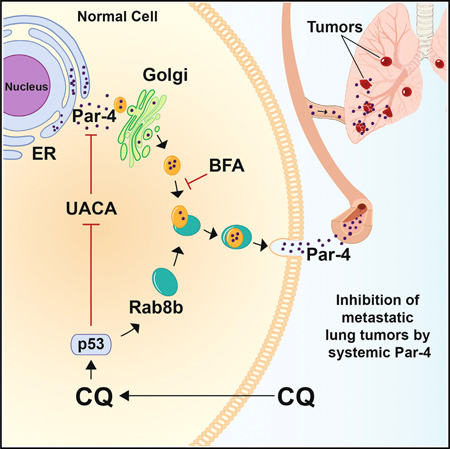

## INTRODUCTION

Among the tumor suppressor proteins expressed ubiquitously by normal cells and tissues, Prostate apoptosis response-4 (Par-4, also known as PAWR) exhibited both intracellular and extracellular pro-apoptotic functions (Hebbar et al., 2012). Studies in cell culture and mouse models indicated that Par-4 selectively induced apoptosis in diverse cancer cells but not in normal cells (Hebbar et al., 2012). Consistent with this finding, transgenic mice overexpressing Par-4 resisted the growth of tumors (Zhao et al., 2007). We and others have shown that inactivation, downregulation, or mutation of Par-4 has occurred in several types of cancers (Hebbar et al., 2012). Par-4 secreted by normal and cancer cells bound selectively to a receptor GRP78 on the cancer cell surface, where it induced apoptosis by a caspase-8/caspase-3-dependent pathway (Burikhanov et al., 2009). In contrast to cancer cells, normal cells expressed low-to-undetectable levels of basal or inducible GRP78 at the cell surface and resisted apoptosis by extracellular Par-4 (Burikhanov et al., 2009).

Par-4 null mice developed spontaneous, as well as inducible, tumors at a higher frequency than that seen in wild-type (Par-4^+/+^) mice (García-Cao et al., 2005), an outcome that implied that basal levels of Par-4 were effective in regulating tumor growth. Elevation of extracellular Par-4 in cell-culture conditioned medium (CM) induced apoptosis of cancer cell cultures (Burikhanov et al., 2009, 2013, 2014a, 2014b), and systemic elevation of Par-4 in mice inhibited growth of tumors (Zhao et al., 2011). Using a chemical biology approach to elevate the secretion of Par-4 from normal cells, we identified a 3-arylquinoline, Arylquin-1, as a potent Par-4 secretagogue in normal cell cultures and mouse models (Burikhanov et al., 2014b). The secreted Par-4, which was produced by the administration of Arylquin-1, induced the paracrine apoptosis of diverse cancer cells (Burikhanov et al., 2014b). To identify other compounds that functioned as Par-4 secretagogues, we tested a panel of FDA (Food and Drug Administration)-approved generic drugs for Par-4 secretion from normal cells. This screening process identified the antimalarial drug chloroquine (CQ) as a potent inducer of Par-4 secretion from normal cells under conditions that showed no normal cell death.

In clinical trials, CQ also showed encouraging results in subsets of diverse cancers (Rebecca and Amaravadi, 2016). CQ induced cytotoxic effects in tumors by blocking autophagy, but in mice containing oncogenic K-ras and lacking functional p53, loss of autophagy accelerated tumor progression (Rosenfeldt et al., 2013). CQ has been reported to display pleiotropic mechanisms of action that include inhibition of autophagy by blocking the fusion of the autophagosome with the lysosome (Boya et al., 2003), lethal lysosomal destabilization (Maycotte et al., 2012), and normalization of tumor vasculature (Maes et al., 2014). Absent from this list, however, was any report of the induction of protein secretion by CQ. The findings reported here suggested that CQ induced Par-4 secretion from normal cells by a mechanism that was dependent on tumor suppressor p53 and its transcriptional target, Rab8b, and that Par-4 was essential for paracrine apoptosis of p53-deficient cancer cells and tumor growth inhibition by CQ. Moreover, CQ-induced secretion of Par-4 was prevented by brefeldin A (BFA), which blocked the conventional pathway but not the non-conventional pathways (Klausner et al., 1992; Grieve and Rabouille, 2011). Loss of Rab8a, which was involved in autophagic secretion (Dupont et al., 2011), did not prevent the induction of Par-4 secretion by CQ. This finding indicated that Par-4 secretion occurred independently of the non-conventional autophagic pathway.

## RESULTS

### CQ Induced Robust Par-4 Secretion from Normal Cells

**Figure 1:**
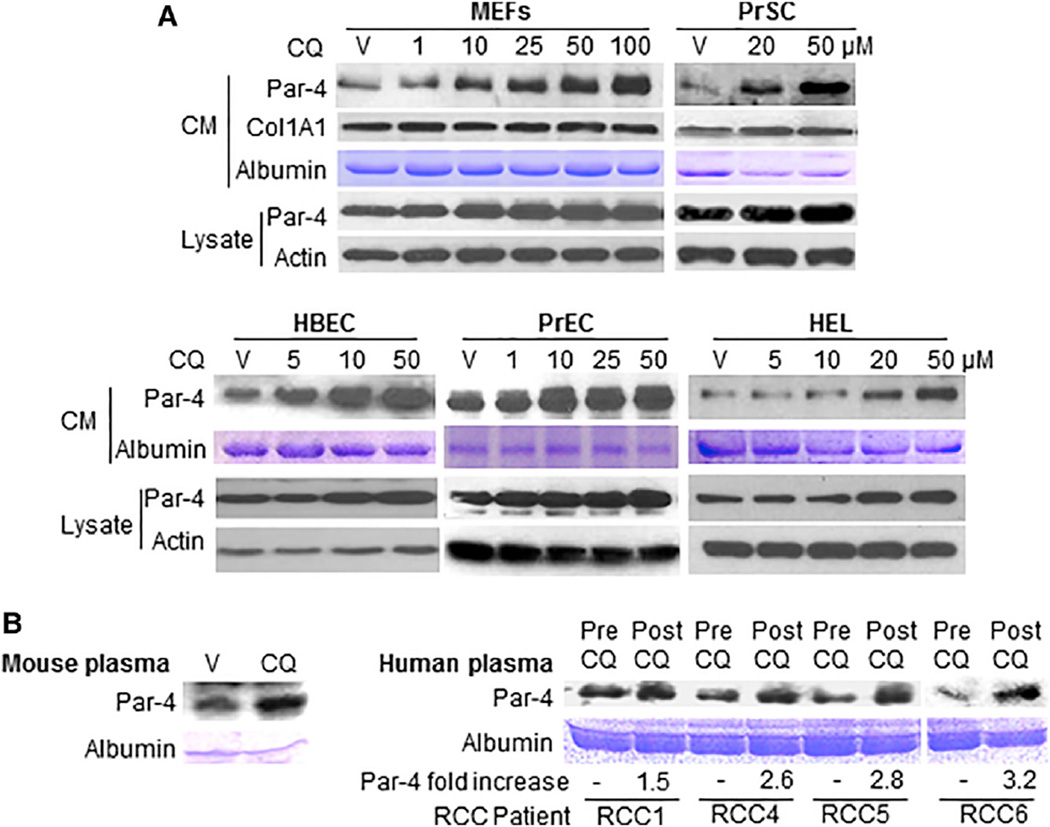
CQ Induced Secretion of Par-4 (A) CQ induced Par-4 secretion in normal cells. Various normal cell lines were treated with the indicated amounts of chloroquine (CQ) or vehicle (V) control for 24 hr. The conditioned medium (CM) and whole-cell lysates were subjected to western blot analysis with the indicated antibodies. We used Collagen1A1 (Col1A1) as loading control for protein secretion and/or albumin levels in plasma as control for CM. Actin served as a loading control for the lysates. PrSC, normal human prostate stromal cells; PrEC, normal human prostate epithelial cells. (B) CQ induced systemic secretion of Par-4 in mice and patients. (Left) C57BL/6 mice were injected i.p. with a single dose (50 mg/kg body weight) of CQ, and plasma samples were collected after 24 hr and processed by western blot analysis. Data are representative of four mice. (Right) RCC patients were treated with HCQ (400 mg/day) pre-operatively for 2 weeks in a CQ clinical trial at the University of Pittsburgh. Pre- and post-treatment plasma (Pre and Post, respectively) were subjected to western blot analysis. Fold increase in post-treatment Par-4 levels relative to the corresponding pre-treatment levels is shown. See also Figure S1.

To test FDA-approved generic drugs for induction of Par-4 secretion, we used a panel of 17 structurally related drugs containing either quinoline or quinolone pharmacophores (Table S1). Mouse embryonic fibroblasts (MEFs) with low (i.e., 4–5) passages were treated with the compounds or vehicle, and Par-4 secretion in the CM was determined by western blot analysis. CQ and hydroxychloroquine (HCQ) induced robust secretion of Par-4 (Figure S1A). To confirm the findings of this initial screen, normal mouse and human cell lines were treated with various concentrations of either CQ or vehicle for 24 hr, and their CM was examined for secreted Par-4. CQ caused dose-dependent secretion of Par-4 in the CM from wild-type (p53^+/+^) MEFs, as well as from normal human prostate stromal cells (PrSCs) and epithelial cells (PrECs) and from normal human lung fibroblast (HEL) cells and epithelial cells (HBECs) (Figure 1A).

CQ blocked autophagic degradation of proteins by preventing the fusion of the autophagosome with the lysosome (Mariño et al., 2014), and we confirmed that CQ functioned as expected and caused the accumulation of LC-3II and p62 (Figure S1B). However, the process of preventing autophagy may trigger caspase activation and apoptosis (Amaravadi et al., 2007). We, therefore, determined whether CQ-induced secretion of Par-4 occurred by an apoptosis-independent process. Wild-type MEF cells were pretreated with the pan-caspase inhibitor zVAD-fmk, which blocked caspase-dependent apoptosis, and then were further treated with either CQ or vehicle. As seen in Figure S1B, CQ-induced secretion of Par-4 was not inhibited by zVAD-fmk, a finding that indicated that CQ-induced Par-4 secretion was not associated with apoptosis of the cells. Normal cells did not undergo cell-cycle arrest or growth inhibition when treated with CQ (Figure S1B). Moreover, there was no difference in p62/SQSTM1 levels between the Par-4^+/+^ and Par-4^−/−^ cells at basal level and after CQ treatment (Figure S1B), implying that Par-4 does not affect autophagy.

We next determined whether CQ induced Par-4 secretion in cancer cells. Lung cancer cells or prostate cancer cells were treated with CQ, and their CM was examined for Par-4 secretion. In contrast to its effects in normal cells, as noted earlier, CQ failed to induce Par-4 secretion in prostate cancer cells (LNCaP, C4-2B, DU145, and PC-3) and in lung cancer cells (H460 and A549) (Figure S1C). To ascertain the physiological relevance of these findings, immunocompetent mice were treated with a single injection of CQ or control vehicle, and, after 24 hr, their plasma samples were tested for systemic levels of Par-4. Relative to vehicle treatment, CQ induced the robust elevation of Par-4 in mouse plasma (Figure 1B). In addition, plasma samples from renal cell carcinoma (RCC) patients—who were taking HCQ prior to surgery in a clinical trial to test the efficacy of repurposing CQ as a single agent against cancer (http://www.clinicaltrials.gov/ct2/show/NCT01144169?term=hydroxychloroquine&rank=10)—showed elevated levels of Par-4 relative to pre-treatment levels (Figure 1B).

### Induction of Par-4 Secretion from Normal Cells by CQ Caused Paracrine Apoptosis in Cancer Cells

**Figure 2:**
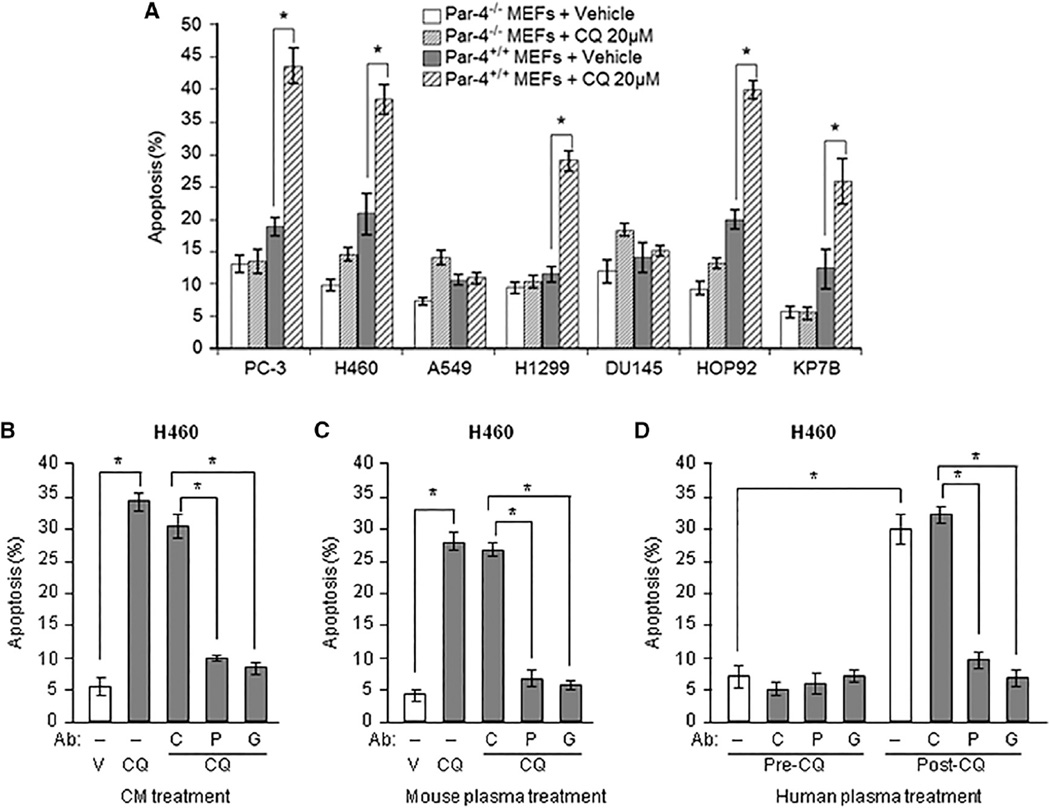
CQ Induced Paracrine Apoptosis of Cancer Cells via Induction of Par-4 Secretion from Normal Cells (A) CQ induced paracrine apoptosis in cancer cells. Par-4^+/+^ or Par-4^−/−^ MEFs were co-cultured with various cancer cells or normal cells and treated with CQ or vehicle. After 24 hr, the cells were scored for apoptosis. (B) CQ induced paracrine apoptosis in cancer cells by a Par-4-dependent mechanism. Aliquots of CM from wild-type MEFs treated with CQ (20 µM) were incubated with control (C) antibody (Ab), Par-4 (P) Ab, or GRP78 (G) Ab and then transferred to H460 cells. After 24 hr, the cells were scored for apoptosis. V, vehicle. (C) Plasma from CQ-treated mice induced apoptosis in cancer cells by a Par-4- and GRP78-dependent mechanism. Plasma from C57BL/6 mice injected with vehicle (V) or CQ was tested for ex vivo apoptosis in H460 cells in the presence of control (C) antibody (Ab), Par-4 (P) Ab, or GRP78 (G) Ab. The cells were scored for apoptosis. (D) Plasma from CQ-treated patients induced apoptosis in cancer cells by a Par-4- and GRP78-dependent mechanism. Aliquots of RCC4 patient plasma were incubated with control (C) antibody (Ab), Par-4 (P) Ab, or GRP78 (G) Ab and then applied to H460 cells. Apoptotic cells were scored after 24 hr. Error bars indicate mean of at least three independent experiments ± SD. *p < 0.0001, by Student’s t test. See also Figure S2.

To determine the biological significance of Par-4 secreted in response to CQ, we tested co-cultures of MEFs and various p53-deficient or p53 wild-type cancer cell lines for apoptosis with CQ or vehicle. The p53-deficient lung cancer cells (H1299, HOP92, and KP7B), as well as the p53-deficient prostate cancer cells (PC-3), and the wild-type p53-expressing lung cancer cells (H460) were sensitive to apoptosis when co-cultured with Par-4^+/+^ MEFs and treated with CQ (Figure 2A). These cells were not sensitive to apoptosis when co-cultured with Par-4^−/−^ MEFs and treated with CQ (Figure 2A). The DU145 (p53-deficient) prostate cancer cells or A549 (p53 wild-type) lung cancer cells, which were resistant to apoptosis by Par-4 (Burikhanov et al., 2009, 2013), were also resistant to apoptosis by the Par-4 secreted from CQ-treated Par-4^+/+^ MEFs in experiments that served as internal controls (Figure 2A). Cancer cell apoptosis was also induced by the CM of Par-4^+/+^ MEFs treated with CQ (Figure 2B). The apoptotic activity of the CM was inhibited by the Par-4 antibody, which neutralized the Par-4 in the CM, or by the GRP78 antibody, which inhibited the binding of Par-4 to its receptor GRP78 on the cancer cell surface (Figure 2B). The A549 cells treated with the nuclear factor κB (NF-κB) inhibitor PS-1145, which elevated the Par-4 receptor GRP78 on the cancer cell surface (Burikhanov et al., 2013), were sensitized to apoptosis by the Par-4 secreted in the CM from MEFs in response to CQ treatment (Figure S2A).

The plasma from mice treated with a single dose of CQ, but not the plasma from control vehicle-treated mice (shown in Figure 1B), also induced ex vivo apoptosis of cancer cells. This activity was, again, neutralized either by the Par-4 antibody or by the GRP78 antibody (Figure 2C). Moreover, the plasma from patient RCC4 showed an induction of Par-4, of ca. 3-fold, following CQ treatment (shown in Figure 1B). Cancer cells (PC-3, H460, HOP92, and H1299) underwent apoptosis with aliquots of post-CQ treatment RCC4 plasma relative to the background level apoptosis produced by aliquots of pre-CQ-treatment plasma (Figure S2B). Importantly, apoptosis of cancer cells by post-CQ treatment plasma from RCC4 was neutralized by the Par-4 or GRP78 antibody (Figure 2D). Taken together, these findings indicated that CQ-induced Par-4 expression levels in either the CM of cell cultures or in the plasma of CQ-treated mice and humans were adequate to trigger apoptosis of cancer cells.

### CQ Induced Paracrine Apoptosis and Tumor Growth Inhibition by Par-4-Dependent Mechanism

**Figure 3:**
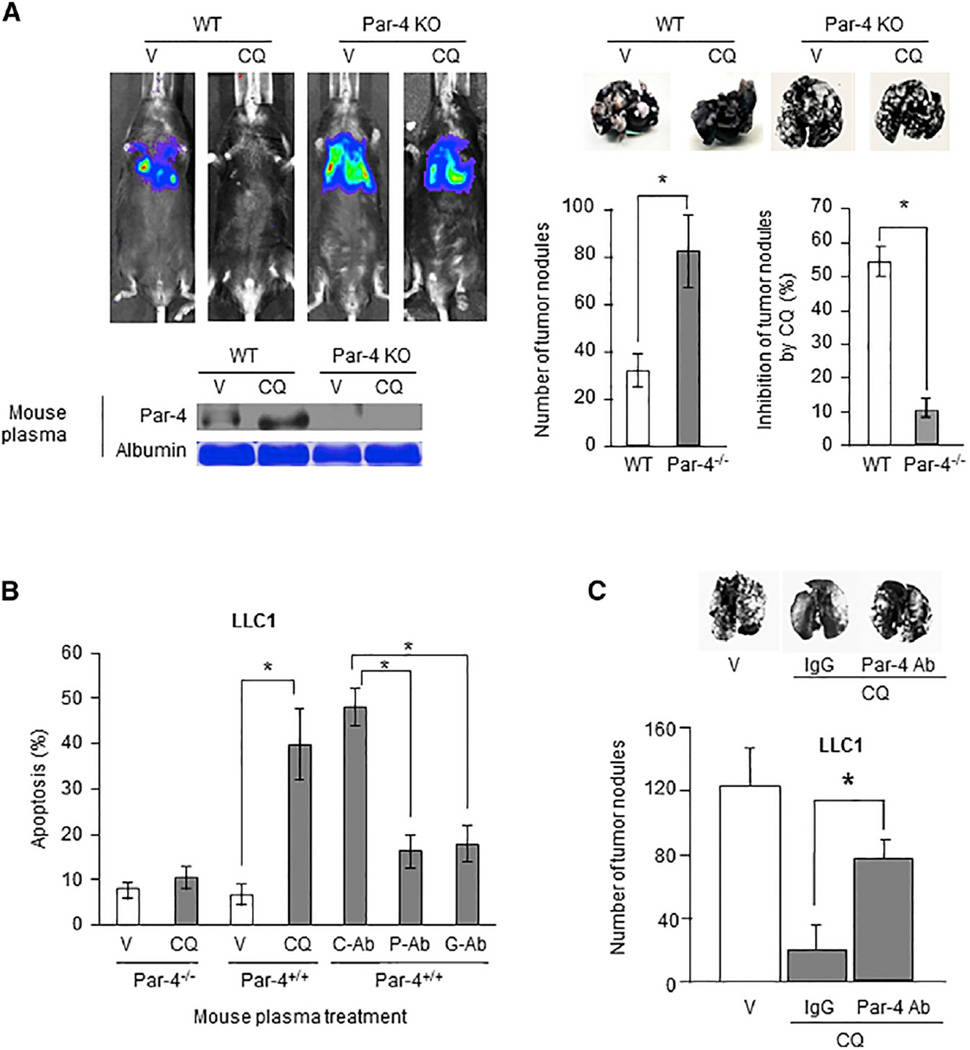
CQ Induced Tumor Growth Inhibition by a Par-4-Dependent Mechanism (A) CQ induced Par-4 secretion and tumor growth inhibition. Wild-type (WT or Par-4^+/+^) or Par-4 knockout (Par-4 KO or Par-4^−/−^) C57BL/6 mice were injected intravenously (i.v.) with LLC1 cells (expressing luciferase) and, 24 hr later, injected i.p. with CQ (25 mg/kg body weight) or vehicle (V) once every day for 5 consecutive days. Plasma from mice was collected 24 hr after the last injection and subjected to western blot (WB) analysis for Par-4 or Coomassie blue staining for albumin (lower left panel). Tumor growth in the mice was followed by fluorescent imaging for luciferase expression using an IVIS imager, and representative images are shown (upper left panel). The lungs were perfused and stained with India ink (upper right panel), tumor nodules were scored, and percent growth inhibition with CQ relative to vehicle was computed (lower right panels). *p < 0.001, by Student’s t test. (B) Par-4 in plasma from CQ-treated mice induced ex vivo apoptosis in LLC1 cells. Aliquots of plasma from Par-4^+/+^ or Par-4^−/−^ mice treated with CQ or vehicle (V), tested by western blot analysis in (A), were incubated with LLC1 cells for 24 hr, and the cells were scored for apoptosis. Moreover, aliquots of plasma from Par-4^+/+^ mice treated with CQ were incubated with control antibody (C-Ab), Par-4 antibody (P-Ab), or GRP78 antibody (G-Ab) and then transferred to LLC1 cells. After 24 hr, the cells were scored for apoptosis. *p < 0.0001, by Student’s t test. (C) CQ-induced Par-4 secretion is essential for tumor growth inhibition. Athymic (*nu*/*nu*) mice were injected intravenously (i.v.) with LLC1 cells and, 24 hr later, injected i.p. with CQ (25 mg/kg body weight) or vehicle once every day for 5 consecutive days. Animals injected with CQ were also injected with either the control IgG or Par-4 polyclonal antibody (Par-4 Ab) (20 µg per injection). After 21 days, the lungs were perfused and stained with India ink (upper panel), and the tumor nodules were scored (lower panel). Error bars indicate mean ± SD. *p = 0.007, by Student’s t test. See also Figure S3.

We previously established that systemic elevation of Par-4 inhibited the growth of LLC1-derived metastatic lung tumor nodules in syngeneic mice (Zhao et al., 2011). In results similar to our findings on the paracrine apoptosis of cancer cells induced by the CM from CQ-treated normal cells (see Figure 2A), we noted that the CM from CQ-treated Par-4^+/+^ MEFs, but not from CQ-treated Par-4^−/−^ MEFs induced apoptosis of LLC1 cells. This apoptotic activity in the CM was inhibited by the Par-4 antibody or GRP78 antibody (Figure S3A). To test whether CQ inhibited the growth of LLC1-derived tumor nodules in this model of experimental metastasis, we injected LLC1 cells intravenously into Par-4^+/+^ (wild-type) or Par-4^−/−^ mice and then injected CQ (intraperitoneally [i.p.], 25 mg/kg body weight) once daily for 5 consecutive days. Tumor growth was examined over a 21-day period. The plasma from CQ-treated Par-4^+/+^ mice, but not from CQ-treated Par-4^−/−^ mice, showed robust Par-4 secretion relative to the plasma from vehicle-treated mice (Figure 3A). Luciferase imaging, as well as the scoring of the tumors in mice treated with vehicle indicated that Par-4^−/−^ mice showed a significantly higher number of lung nodules than the number of nodules in the Par-4^+/+^ mice (Figure 3A). CQ induced >50% inhibition of tumor nodules in the lungs of Par-4^+/+^ mice but only ca.10% inhibition of tumor nodules in Par-4^−/−^ mice (Figure 3A).

The plasma from CQ-treated Par-4^+/+^ mice, but not the plasma from CQ-treated Par-4^−/−^ mice, induced ex vivo apoptosis in LLC1 cells. This activity of the plasma was neutralized by the Par-4 or GRP78 antibody but not by a control antibody (Figure 3B). These results suggested that basal levels of Par-4 in wild-type mice prevented the establishment and growth of cancer cells and that Par-4 levels elevated in response to CQ were essential for the further inhibition of lung tumor nodules.

To test directly the role of Par-4 secreted in response to CQ, we used the Par-4 neutralizing antibody and examined whether it blocked CQ-induced inhibition of LLC1-derived lung tumor nodules. Athymic *nu*/*nu* mice were injected with LLC1 cells intravenously, and after 24 hr, the mice were injected with vehicle or CQ (i.p., 25 mg/kg body weight) once daily for 5 consecutive days. Mice injected with CQ were also injected with either the Par-4 antibody or immunoglobulin G (IgG) control antibody. As seen in Figure 3C, relative to vehicle control, CQ induced >85% inhibition of LLC1 tumor nodules in the lungs of nude mice in the presence of control IgG, but only ca. 40% inhibition of lung tumor nodules in the presence of Par-4 antibody. CQ also inhibited lung metastasis by EO771 breast cancer cells in Par-4^+/+^ mice but not in Par-4^−/−^ mice (Figure S3B). Importantly, CQ inhibited lung metastasis by EO771 cells in the presence of control IgG antibody but not in the presence of Par-4 antibody (Figure S3B). These results indicated that CQ-inducible secretion of Par-4 plays a significant role in inhibition of lung tumor nodules in two different experimental metastasis models. By contrast, yet consistent with the findings of Xia et al. (2014), CQ failed to inhibit the growth of LLC1- or H460-derived tumor xenografts in the flanks of mice (Figure S3C). Collectively, these findings indicated that Par-4 levels secreted by normal cell cultures and from mice and patients in response to CQ were adequate to cause ex vivo apoptosis of cancer cells and to inhibit the growth of metastatic lung tumor nodules in mice.

### Induction of Par-4 Secretion by CQ Required Components of the Classical Secretory Pathway

**Figure 4:**
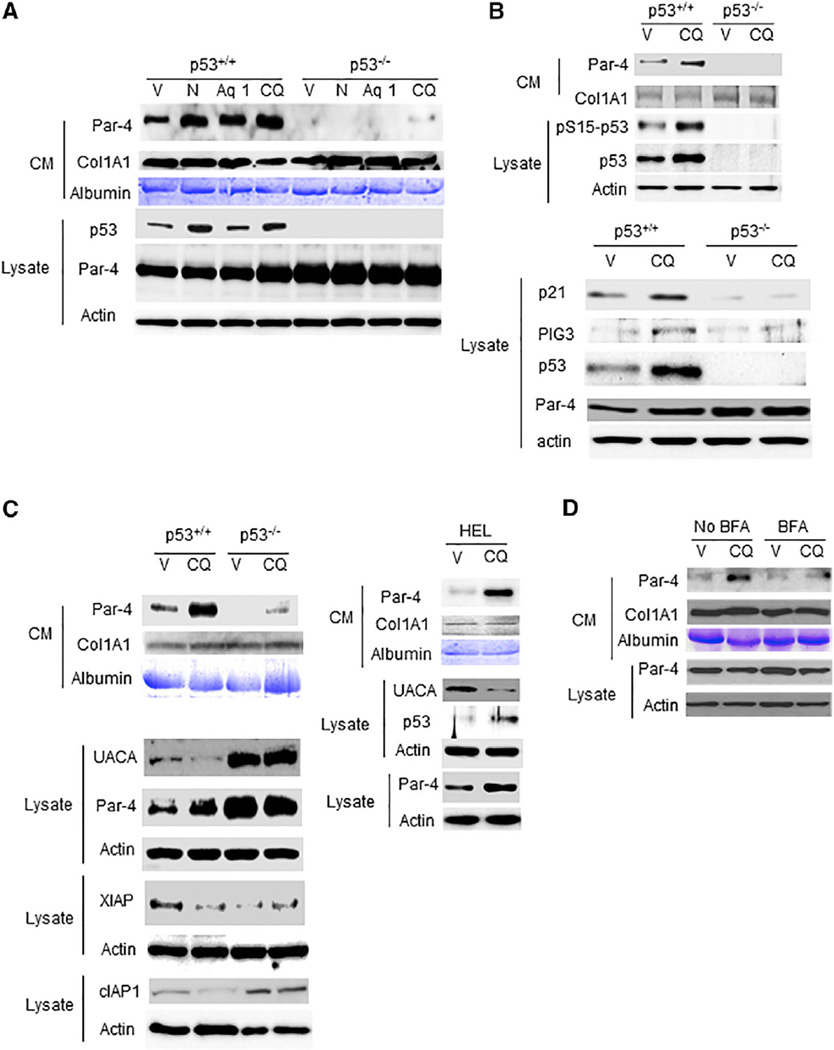
CQ Induced Par-4 Secretion by a BFA-Sensitive, p53-Dependent Pathway (A) CQ induced Par-4 secretion by a p53-dependent mechanism. P53^+/+^ or p53^−/−^ MEFs were treated with CQ (25 µM), Arylquin-1 (Aq 1; 500 nM), Nutlin-3a (N; 10 µM), or vehicle (V) for 24 hr, and the CM and lysates were examined by western blot analysis with the indicated antibodies. (B) CQ induced p53 activation. Wild-type (p53^+/+^) or p53^−/−^ MEFs were treated with CQ (25 µM) or vehicle (V) for 24 hr, and the CM and lysates were examined by western blot analysis with the indicated antibodies. (C) CQ inhibited UACA expression. Wild-type MEFs and p53^−/−^ MEFs (left panel) or HEL cells (right panel) were treated with CQ (25 µM) or vehicle (V) for 24 hr, and the CM and lysates were examined by western blot analysis with the indicated antibodies. (D) CQ induced Par-4 secretion by a BFA-sensitive mechanism. MEFs were pre-treated with BFA (1 µg/mL) or vehicle for 30 min and further treated with CQ (25 µM) or vehicle (V) for 4 hr. The CM and lysates were examined by western blot analysis with the indicated antibodies. See also Figure S4.

We reported previously that Nutlin-3a activated p53 and promoted Par-4 secretion via the BFA-sensitive classical secretory pathway involving the transport of proteins from the endoplasmic reticulum (ER) to the Golgi and onward to the plasma membrane (Burikhanov et al., 2014a). Importantly, p53 function was essential for secretion of Par-4 via the classical pathway (Burikhanov et al., 2014a). As CQ was known to cause activation of p53 through the ATM-signaling pathway (Sohn et al., 2002; Loehberg et al., 2007; Maclean et al., 2008), we tested whether p53 function was required for CQ-induced Par-4 secretion. Wild-type or p53 null MEFs were treated with CQ, Arylquin-1, or Nutlin-3a. As expected, Nutlin-3a, an inhibitor of MDM2 binding to p53 (Vassilev et al., 2004), induced Par-4 secretion in a p53-dependent manner (Figure 4A). Similarly, CQ and Arylquin-1 caused robust induction of Par-4 secretion in wild-type MEFs, but not in p53 null MEFs (Figure 4A). Moreover, CQ induced p53 activation, as judged by the increase in phospho-S15-p53 and total p53 expression in wild-type MEFs (Figure 4B). Activation of p53 by CQ was accompanied by increased p53-dependent transcription (Figure S4A) and upregulation of p53-responsive genes p21 and PIG3 in MEFs and normal mouse lung tissues (Figures 4B and S4B). Although CQ caused an increase in total Par-4 protein levels (see Figures 1 and 4), it did not induce Par-4 at the RNA level (Figure S4C), implying that Par-4 protein levels were elevated by CQ-induced translational/posttranslational regulation. Collectively, these data suggested that p53 was activated by CQ and that p53 function was required for induction of Par-4 secretion by CQ but not for elevation of intracellular Par-4 protein levels. These data are consistent with chromatin immunoprecipiation (ChIP) data (Figure 6B) and an observation that the *Par-4* (*PAWR*) gene lacks p53 consensus-binding sites for transcriptional regulation by p53 (Burikhanov et al. (2014a).

We previously showed that UACA, an inflammation-associated protein, bound to Par-4, sequestered it in the ER, and thereby prevented Par-4 secretion (Burikhanov et al., 2013). UACA expression was inhibited by p53 but was induced by NF-κB activation. Activation of p53 or inhibition of NF-κB activation promoted Par-4 secretion (Burikhanov et al., 2013). Because CQ activated p53, we tested whether CQ suppressed NF-κB activity and UACA expression. Normal and cancer cells were transfected with reporter controls for NF-κB or AP-1 and treated with CQ or vehicle for 18 hr. CQ inhibited NF-κB activity, but not AP-1 activity, in normal cells (Figure S4D). Moreover, CQ inhibited the expression of UACA and NF-κB-regulated genes cIAP1 and XIAP in a p53-dependent manner (Figure 4C), and BFA inhibited CQ-induced Par-4 secretion (Figure 4D). By contrast, in cancer cells that failed to show induced Par-4 secretion in response to CQ (Figure S1C), CQ neither induced p53 nor inhibited NF-κB activity, and CQ consistently failed to downregulate UACA (Figures S4D and S5A). Collectively, these studies indicated that, similar to our findings with Nutlin-3a (Burikhanov et al., 2014a), CQ induced Par-4 secretion by the classical secretory pathway that is dependent on UACA suppression by p53 and by inhibition of NF-κB activity.

### Rab8b Is a Target of p53 Critical for Par-4 Secretion from Normal Cells

**Figure 5:**
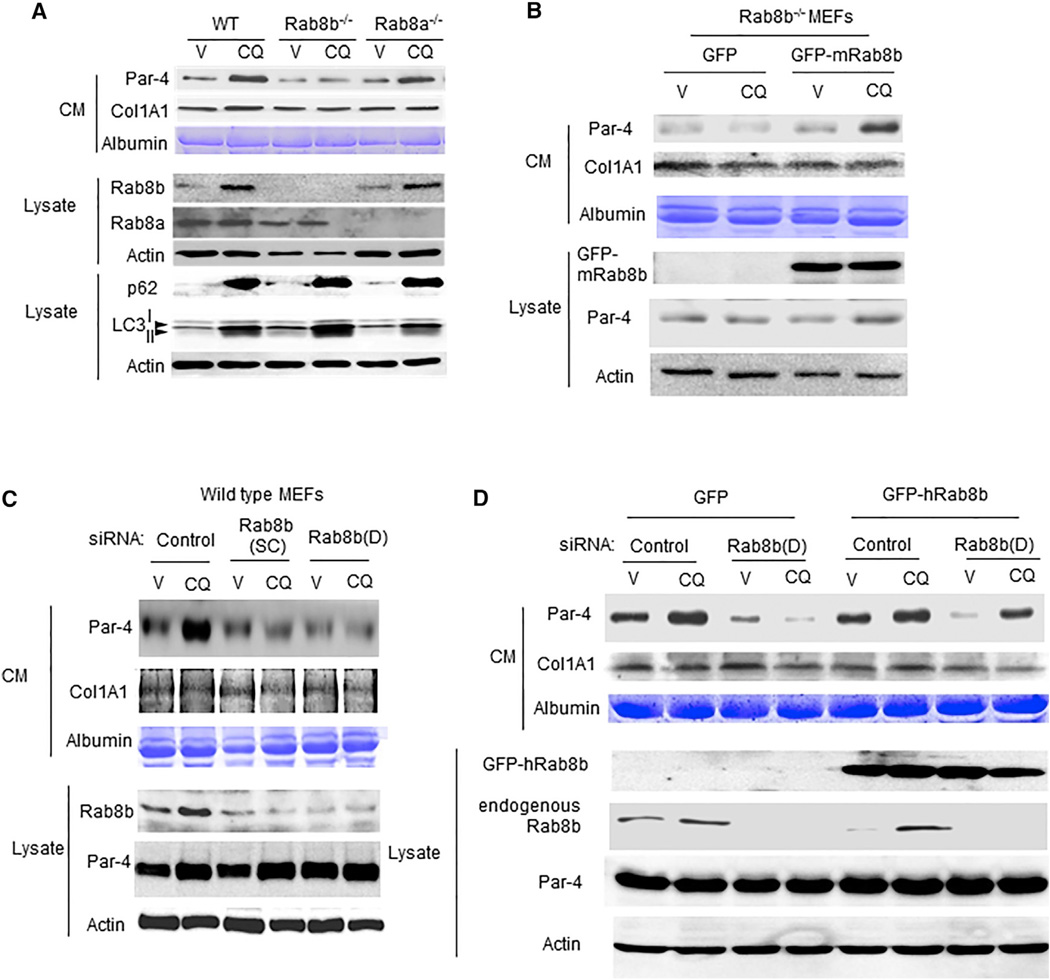
CQ Induced Par-4 Secretion Is Dependent on Rab8b (A) CQ induced Par-4 secretion by a Rab8b-dependent mechanism. Rab8 wild-type (WT), Rab8b^−/−^, or Rab8a^−/−^ MEFs were treated with CQ (25 µM) or vehicle (V) for 24 hr, and the CM or lysates were examined by western blot analysis with the indicated antibodies. (B) Induction of Par-4 secretion in response to CQ in Rab8b null cells was restored by re-introduction of Rab8b. Rab8b null MEFs were transiently transfected with GFP-mouse Rab8b (GFP-mRab8b) expression construct or GFP-expression construct for control, and the transfectants were treated with CQ (25 µM) or vehicle for 24 hr. The CM and lysates from the cells were examined by western blot analysis with the indicated antibodies. (C) Par-4 secretion in response to CQ was inhibited by Rab8b siRNAs. Wild-type MEFs were transfected with siRNA duplexes from two different sources, Dharmacon (D) and Santa Cruz Biotechnology (SC), or with scrambled siRNA duplexes for control, and the transfectants were treated with CQ (25 µM) or vehicle for 24 hr. The CM and lysates from the cells were examined by western blot analysis with the indicated antibodies. (D) Introduction of human Rab8b in Rab8b-knockdown MEFs resulted in restoration of Par-4 secretion in response to CQ. MEFs were transfected with the indicated siRNAs, 24 hr later, they were re-transfected with GFP-human Rab8b (GFP-hRab8b) or GFP expression construct, and the transfectants were treated for 24 hr with CQ (25 µM) or vehicle. Western blot analysis of the CM and lysates was performed by using the indicated antibodies. Knockdown of endogenous Rab8b was confirmed with the Rab8b antibody, and expression of GFP-hRab8b was detected with the GFP antibody. See also Figure S5.

UACA suppression was essential for induced secretion of Par-4 in response to Nutlin-3a. UACA suppression by RNAi resulted in induced secretion of Par-4 only from cells expressing wild-type p53, but not from cells lacking p53 expression (Burikhanov et al., 2014a). This finding indicated that p53 function was critical for Par-4 secretion and that p53 must regulate other components of the classical secretory pathway to induce Par-4 secretion. Members of the Rab family of GTPases were essential for vesicle transport of proteins from the ER to the Golgi and from the Golgi to the plasma membrane for secretion (Stenmark and Olkkonen, 2001). Several members of the Rab family contained putative, p53 consensus-binding sites in their promoter region (http://www.sabiosciences.com/chipqpcrsearch.php). Because Par-4 was localized to the apical surface of prostate secretory luminal cells (Boghaert et al., 1997), and because recent studies indicated that several Rab proteins were involved in apical transport of proteins (Sobajima et al., 2014; Sato et al., 2014), we tested whether the expression of *Rab* genes containing putative p53-binding sites in their promoter region were essential for Par-4 secretion. These studies indicated that Rab8b null MEF cells, but not Rab8a null MEF cells, failed to show induction of Par-4 secretion by CQ (Figure 5A). CQ-induced Rab8b expression, but not Rab8a expression, in wild-type MEFs (Figure 5A). CQ also induced Rab8b in normal human cells (Figure S5B) but not in cancer cells (Figure S5A). We confirmed that Rab8b null MEF cells were not deficient in p53 induction in response to CQ (Figure S5C). Moreover, we tested the effect of transiently transfecting Rab8b null MEFs with mouse Rab8b expression construct or vector for control and noted that CQ induced Par-4 secretion in Rab8b reconstituted cells (Figure 5B). Knocking down Rab8b with small interfering RNAs (siRNAs) resulted in the inhibition of Par-4 secretion in the CM, as judged by western blot analysis (Figure 5C), and the reintroduction of GFP-Rab8b into MEFs, after knockdown of endogenous Rab8b with siRNA rescued Par-4 secretion (Figure 5D).

**Figure 6:**
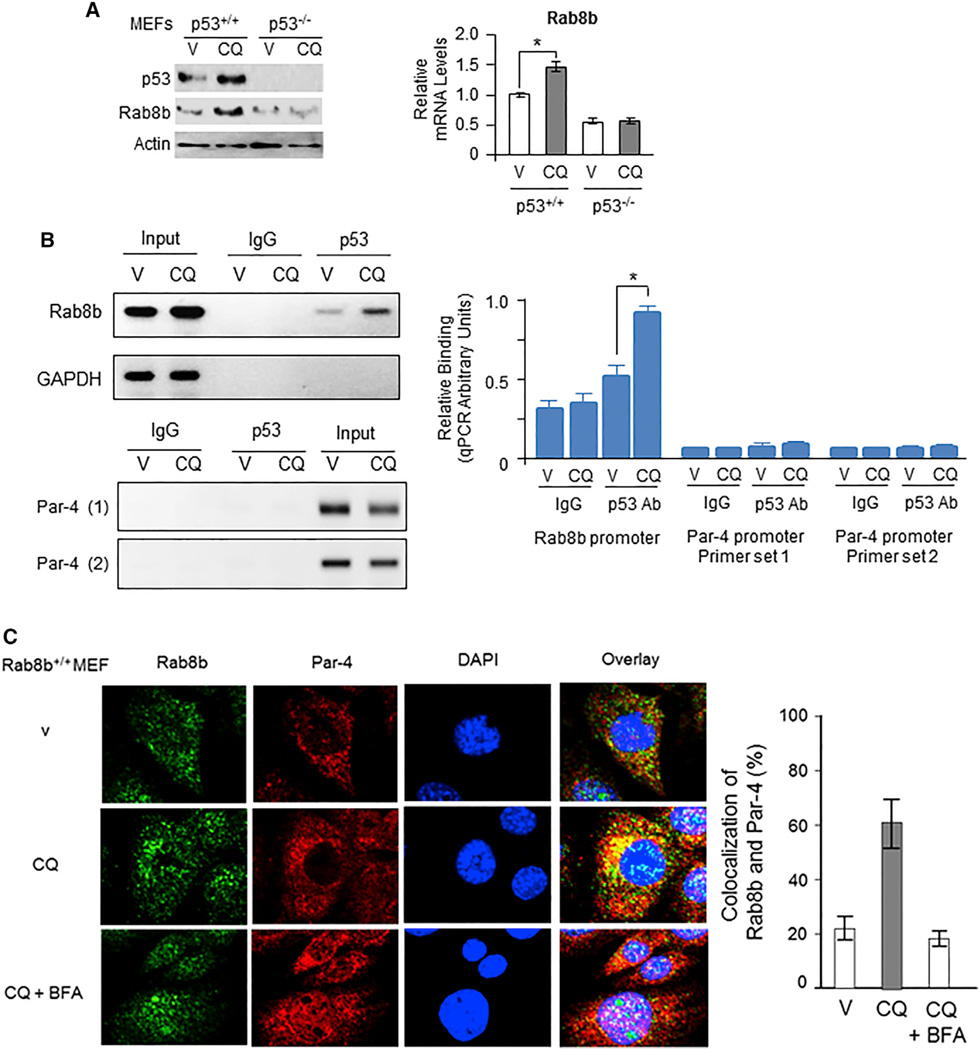
Rab8b Is a Direct Downstream Target of p53, which Is Activated by CQ (A) CQ induced Rab8b protein and mRNA levels in a p53-dependent manner. Wild-type (p53^+/+^) or p53^−/−^ MEFs were treated with CQ (25 µM) or vehicle (V) for 24 hr; and either the lysates were examined by western blot analysis with the indicated antibodies (left panel), or mRNA prepared from the cells was examined by real-time qRT-PCR (right panel). *p < 0.001, by Student’s t test. (B) p53 directly bound to its consensus-binding site in the *Rab8b* promoter. MEF cells were treated with CQ or vehicle (V) and subjected to ChIP analysis with p53 antibody (p53 Ab) or control IgG antibody, and immunoprecipitated DNA fragments were amplified and analyzed on agarose gels (left panel) or by real-time qPCR (right panel) with primers near the p53-binding site in Rab8b promoter. The immunoprecipitated fragments were similarly analyzed with random primers for GAPDH promoter or two different primer sets for the Par-4 gene, which does not contain a p53-binding site. *p < 0.001, by Student’s t test. (C) Par-4 co-localized with Rab8b+ vesicles in CQ-treated cells. MEFs were treated with vehicle (V) or CQ (25 µM) in the absence or presence of BFA (1 µg/mL) for 24 hr and subjected to ICC analysis for Par-4 (red fluorescence) and Rab8b (red fluorescence). Cells were stained with DAPI to reveal their nuclei (blue fluorescence). Co-localization of Par-4 and Rab8b vesicles in the overlay images is indicated by yellow fluorescence. Note the dissociation of Par-4 and Rab8b (loss of yellow fluorescence but retention of red and green fluorescence) in the CQ + BFA panel. Cells showing co-localization of Rab8b and Par-4 were scored, and the data are expressed as percentage of cells showing co-localization (right panel). Error bars indicate mean of at least three independent experiments ± SD. See also Figure S6.

The guardian of the genomep53 binds to a consensus motif 5′-PuPuPuC4(A/T)(A/T) G7PyPyPy [0–13] PuPuPuC4(A/T)(A/T)G7PyPyPy-3′ (where purines at positions 4 and 7 are critical) in the promoter of genes that it transcriptionally regulates (el-Deiry et al., 1992). Putative p53-binding sites 5′-GGGGCACGCCCGAGGCTCCCC-3′ on human chromosome 15 (corresponding to nucleotides at positions 63481874 to 63481894) and 5′-GGAGAGCCGCGGGCGTGCCT-3′ on mouse chromosome 9 (corresponding to nucleotides at positions 66767671 to 66767690) were noted in the promoter regions upstream of the transcription start site of human and mouse *Rab8b*, respectively (http://www.sabiosciences.com/chipqpcrsearch.php). CQ enhanced Rab8b protein levels in p53 wild-type cells, but not in p53 null cells, and quantitative real-time PCR studies indicated that CQ induced Rab8b mRNA in a p53-dependent manner (Figure 6A).

To determine whether p53 directly binds to this consensus-binding motif within the Rab8b promoter in order to induce Rab8b expression, we treated MEF cells with CQ or vehicle and subjected the DNA to chromatin immunoprecipitation analysis with p53 antibody (Ab) or control IgG antibody. As seen in Figure 6B, relative to vehicle, treatment with CQ resulted in elevated levels of chromatin immunoprecipitation of the p53-consensus motif in Rab8b. By contrast, either the control IgG antibody used along with primers near the p53-binding site in the Rab8b promoter or the p53 antibody used along with primers corresponding to the region in the GAPDH promoter or Par-4 gene, where p53 was not expected to bind, failed to show chromatin immunoprecipitation (Figure 6B). These findings indicated that p53 directly bound toRab8b and that CQ treatment resulted in increased interaction of p53 with its binding motif in the Rab8b promoter.

We also performed an immunocytochemical (ICC) analysis to determine the localization of Par-4 in Rab8b-coated vesicles. Relative to vehicle treatment, CQ induced an increase in the co-localization of Par-4 with Rab8b-containing vesicles, and BFA prevented Par-4 from entering the Rab8b vesicles (Figures 6C and S6). Together, these findings indicated that the *Rab8b* gene was induced in a p53-dependent manner and that Rab8b was essential for CQ-induced secretion of Par-4 by the BFA-sensitive post-Golgi pathway to plasma membrane.

## DISCUSSION

An unbiased screen of FDA-approved quinoline- and quinolone-containing drugs as Par-4 secretagogues led to the identification of the anti-malarial drug CQ and its hydroxylated analog, HCQ, as robust inducers of Par-4 secretion from normal cells. CQ also induced p53 expression and required p53 for successful induction of Par-4 secretion. Our studies indicated that p53 induced by CQ directly bound to the promoter of Rab8b and promoted Par-4 secretion through the upregulation of Rab8b, a previously unknown target of p53. Importantly, Rab8b expression was critical for Par-4 secretion. CQ-induced secretion of Par-4 was prevented by BFA, which blocked the conventional pathway by inhibiting the post-Golgi transport of the protein cargo to the plasma membrane but which did not block the non-conventional pathways (Klausner et al., 1992; Grieve and Rabouille, 2011). Loss of Rab8a, which was necessary for autophagic secretion (Dupont et al., 2011), did not prevent the induction of Par-4 secretion by CQ, and Par-4 secretion occurred independently of the non-conventional, autophagic pathway. The physiological relevance of the findings was immediately evident, because CQ induced elevated levels of Par-4 secretion systemically in mice and patients. These Par-4 levels were sufficient to induce paracrine apoptosis of diverse p53-deficient cancer cells. It was particularly noteworthy that CQ inhibited the growth of lung tumor nodules in a Par-4-dependent manner. Collectively, CQ induced tumor cell apoptosis and inhibition of lung tumor nodules via a paracrine effect of Par-4 secreted from normal cells. Because CQ showed encouraging results in clinical trials designed to test the potential repurposing of CQ for cancer treatment (Rebecca and Amaravadi, 2016), and because Par-4 induced apoptosis in a broad range of cancer cells, but not in normal cells (Burikhanov et al., 2009), the tumor-growth-inhibitory action of CQ was attributed to its ability to induce Par-4 secretion from normal cells.

### Structural Similarities of CQ with Arylquin-1

CQ and Arylquin-1 shared a common pharmacophore—namely, the heterocyclic quinoline ring—but they differed in two important respects. First, they differ appreciably at the C-3 and C-4 positions, where CQ possessed a dibasic, 5-(N,N-diethylamino) pentyl-2-amino subunit at C-4 and where Arylquin-1 possessed a non-basic, *ortho*-fluorophenyl subunit at C-3. A second difference between these two secretagogues lies in the presence of the C-7 chloro group in CQ and the C-7 N,N-dimethylamino group in Arylquin-1. The second difference between CQ and Arylquin-1 appeared to be of less consequence than the primary difference at C-3 and C-4. Although both agents promoted Par-4 secretion, and both possessed a common quinoline substructure, it would be premature to conclude that one is a surrogate for the other, given the differences in basicity and hydrophobicity at C-3 and C-4 that could alter their individual mechanisms of action.

### CQ Induced p53 Activation and Par-4 Secretion

CQ blocked degradative autophagy at the final step of autophagosome fusion with the lysosome and did not promote secretory autophagy involving the inflammasome and Rab8a vesicles (Dupont et al., 2011). Our studies indicated that CQ induced the accumulation of p62 and LC3II in normal cells. This finding implied that CQ blocked the expected autophagy. However, CQ has multiple modes of action in cells, and prior studies suggested that CQ-induced activation of ATM results in activation and elevated expression of p53 that, in turn, triggered the apoptosis of selective cancer cells (Loehberg et al., 2007; Maclean et al., 2008). In the present study, CQ showed activation of p53 in normal cells; and, similar to our findings with Nutlin-3a, which was structurally unrelated to CQ but also activated p53 by inhibition of MDM2 (Burikhanov et al., 2014a), the growth of normal cells was not inhibited following p53 activation by CQ. On the contrary, CQ-induced activation of p53 stimulated normal cells to secrete the cancer-selective, pro-apoptotic, Par-4 protein. The ability of p53 to induce the secretion of proteins represented a previously unrecognized function for p53 (Burikhanov et al., 2014a; Yu et al., 2006). Unlike most proteins that lacked a leader sequence and whose secretion was induced by p53 via the non-conventional, exosomal pathway (Yu et al., 2006), p53 induced the secretion of Par-4 via the conventional secretory pathway (Burikhanov et al., 2014a).

Although CQ was reported to activate multiple processes in cells, p53-dependent induction of Par-4 secretion by CQ was functionally linked to the ability of Par-4 to induce paracrine apoptosis of cancer cells and inhibit the growth of lung tumor nodules in mice. Accordingly, in response to CQ, p53 null cells failed to secrete Par-4, and Par-4 null cells or Par-4 null mice failed to show paracrine apoptosis or tumor growth inhibition, respectively. It was interesting that CQ failed to induce robust Par-4 secretion in a broad range of cancer cells. The inability to secrete Par-4 was associated with the lack of p53 induction by CQ. Thus, CQ selectively induced Par-4 secretion from normal cells by a mechanism tightly linked to p53 activation. The studies reported here, thus, uncovered a previously undiscovered function of CQ to induce protein secretion via p53 activation.

### CQ Induced Par-4 Secretion by Rab8b-Dependent Mechanism

Although the transcription of proteins with diverse functions was undoubtedly regulated by p53, the downstream mediators of p53-dependent trafficking of proteins for secretion via the conventional secretory pathway remained unclear at the outset of this work. Our previous studies (Burikhanov et al., 2014a) indicated that Nutlin-3a induced direct binding of p53 to the promoter of the UACA gene at the consensus-binding motif and inhibited its expression. This binding event allowed the release of Par-4, which was sequestered by UACA in the ER, for secretion. The present study showed that CQ-induced Par-4 secretion in normal cells was also associated with inhibition of NF-κB activity and its downstream target UACA. By contrast, NF-κB activity and UACA expression were not inhibited by CQ in cancer cells, and this blockade presumably prevented Par-4 secretion from those cells in response to CQ. Unlike CQ and Nutlin-3a, which inhibited UACA expression, Arylquin-1 did not inhibit UACA (Figure S5D). As Arylquin-1 caused the release of Par-4 from vimentin for secretion (Burikhanov et al., 2014b), these findings indicated that Par-4 secretagogues induced Par-4 secretion by either a UACA-suppression-dependent or a UACA-suppression-independent mechanism.

Although UACA downregulation was essential for Par-4 secretion, it was not sufficient for Par-4 secretion in normal cells lacking p53 function (Burikhanov et al., 2014a). We, therefore, sought to identify other regulators of Par-4 secretion whose function was dependent on p53. In particular, we focused on GTPase-driven protein transport vesicles, because they were critical for trafficking proteins from the Golgi to the plasma membrane. These studies identified Rab8b as a critical mediator of Par-4 secretion by CQ. Although it was conceivable that p53 regulated still other protein transporters along the conventional pathway, our studies indicated that Rab8b met the key essential criteria for Par-4 transport for secretion in response to CQ: (1) a p53-binding motif was present in Rab8b, but not in the closely related isoform Rab8a, and, accordingly, only Rab8b was induced by CQ in a p53-dependent manner; (2) p53 directly bound to the promoter of Rab8b at its consensus-binding motif; (3) Rab8b null MEFs showed p53 induction in response to CQ but failed to induce Par-4 secretion; (4) all the currently known Par-4 secretagogues— namely, Nutlin-3a, Arylquin-1, and CQ—induced Par-4 secretion by a Rab8b-dependent mechanism (Figure S5E); (5) Par-4 was expressed at the apical surface of luminal cells, as seen, for instance, in the secretory epithelial cells of the prostate (Boghaert et al., 1997), and Rab8b was essential for the apical transport of proteins (Sato et al., 2014); (6) Par-4 co-localized with Rab8b vesicles in normal cells, and co-localization was inhibited by BFA; and (7) cancer cells that failed to induce p53 and Rab8b expression also failed to show induction of Par-4 secretion in response to CQ. Collectively, these findings identified Rab8b as a target of p53 that was essential for Par-4 transport from the Golgi to the plasma membrane via the BFA-sensitive classical secretory pathway. Because Rab8b null cells failed to induce Par-4 secretion in response to CQ, Rab8b was absolutely necessary for CQ-induced Par-4 secretion. Studies are underway to evaluate the significance of other Rabs in the action of CQ and to determine whether the GTPase function of Rab8b was involved in the sorting of Par-4 into transport vesicles in the post-Golgi trafficking that led to secretion.

### Paracrine Apoptosis and the Tumor Growth Inhibitory Action of CQ Were Dependent on Par-4

Previously known functions of CQ include autophagy regulation, lysosomal destabilization, and normalization of tumor vasculature. This study uncovered the ability of CQ to induce the secretion of Par-4, a pro-apoptotic tumor suppressor from normal cells. Both human and mouse fibroblasts and epithelial cells of diverse origins showed Par-4 secretion, and Par-4 secretion was observed from normal mice, as well as from human renal cancer patients who were treated with CQ in a clinical trial. Moreover, CQ-induced Par-4 secretion was sufficient to effect the ex vivo apoptosis of cancer cells. CQ inhibited the growth of lung tumor nodules in wild-type mice but not in Par-4 null mice, an observation that implied that Par-4 function was critical for CQ-induced lung tumor growth inhibition. Because our previous studies indicated that Par-4, when injected in mice, inhibited the growth of lung tumors (Zhao et al., 2011), and because the plasma of CQ-treated mice induced ex vivo apoptosis by a Par-4-dependent mechanism, Par-4 secretion induced in response to CQ was involved in the experimental metastasis inhibitory effect of this FDA-approved drug. CQ showed encouraging results in clinical trials designed to re-purpose it as an anti-cancer drug, especially in combination with standard-of-care therapy (Rebecca and Amaravadi, 2016). The present findings indicated that the mechanism of CQ action was not limited to its direct cytotoxic action via autophagy inhibition and that Par-4 was a significant contributor to the paracrine effects of CQ against metastatic lung tumor growth.

In summary, this work identified the mechanism by which CQ caused paracrine apoptosis and tumor growth inhibition. This mechanism involved induction of Par-4 secretion by CQ from normal cells at levels sufficient to cause the paracrine apoptosis of cancer cells. Our studies revealed an unrecognized role for Rab8b, a target of p53, in trafficking Par-4 for secretion. Secretatogues such as CQ utilized p53 activation, which, in turn, induced Rab8b for Par-4 transport via the classical secretory pathway from the Golgi to the plasma membrane for secretion. Thus, although p53 loss in tumors often rendered them refractory to treatment, the relatively safe, FDA-approved drug, CQ, empowered normal cells to secrete Par-4 and induce apoptosis in tumor cells that lacked p53 tumor suppressor function.

## EXPERIMENTAL PROCEDURES

### Cells and Plasmids

Human lung cancer cells A549, H460, H1299, and HOP92; mouse lung cancer LLC1 cells; human prostate cancer cells LNCaP, DU145, and PC-3; and HELs, HBECs, and BEAS-2B cells were from ATCC. C4-2B cells were from Leland Chung (Cedars-Sinai Medical Center), and normal human PrSs and PrEs were from Lonza. Mouse mammary tumor cells EO771 were a kind gift of Linda Matheny-Barlow, Wake Forest Cancer Center, Winston-Salem, NC. KP7B cells were from Tyler Jacks, MIT. Rab8b^+/+^, Rab8a^−/−^, and Rab8b^−/−^ MEFs were derived from wild-type or knockout mice (Sato et al., 2014).

### Antibodies and siRNA Duplexes

Par-4 (R334) for western blot and Par-4 (C-19, sc-1249 goat) for ICC analysis; and GRP78 (N20), Col1A1 (H-197), LC3 (H-47), p21 (C-19), PIG3 (H-300), c-IAP1 (H-83), XIAP (A-7), and pan-cytokeratin (C11) antibodies were from Santa Cruz Biotechnology. UACA, Rab8b (ab124356, rabbit), and Rab8a (ab188574, rabbit) antibodies were from Abcam. Active caspase-3 antibody (Asp175) (5A1E), p62 antibody (Sequestosome-1, SQSTM1) (5114), and p53 antibody (1C12) were from Cell Signaling. The β-actin antibody was from Sigma Chemical. Rab8b siRNA duplexes were from Dharmacon (D), and pools of Rab8b siRNA and scrambled siRNA duplexes were from SantaCruz Biotechnology (SC). Human GFP-tagged Rab8B (RG204651) and mouse GFP-tagged Rab8b (MG202204) were from Origene Technologies.

### Reporter Assays, Cell-Cycle Analysis, and Apoptosis Assays

To determine the effect of CQ on p53-dependent transcription or NF-κB-dependent transcription, cells were co-transfected with p53-response elementluciferase reporter PG13-luc or mutant response element-luciferase reporter MG15-luc (from Addgene) or with NF-κB-luciferase reporter construct, respectively, or with minimal promoter-luciferase vector pGL3 as control, along with β-galactoside construct, and then treated with CQ or vehicle. After 24 hr, cell lysates were quantified for luciferase activity, which was normalized relative to the corresponding β-galactoside activity, as described previously (Burikhanov et al., 2009). Cell-cycle distribution was accessed using propidium iodide staining of wild-type or p53 null MEFs treated with CQ or vehicle in a Becton Dickinson LSRII flow cytometer.

Apoptotic cells were identified by ICC analysis for active caspase-3, and apoptotic nuclei were revealed by 4, 6-diamidino-2-phenylindole (DAPI) staining (Burikhanov et al., 2014b). A total of three independent experiments were performed, and approximately 500 cells were scored in each experiment for apoptosis under a fluorescent microscope.

### Quantitative Real-Time RT-PCR Assay

Wild-type MEFs and p53 null MEFs were treated with either vehicle or CQ (25 µM), and RNA was purified using the RNeasy Plus Kit (QIAGEN). First-strand cDNA was prepared by reverse transcription using the iScript Select cDNA Synthesis Kit (Bio-Rad), and qPCR was performed using the CFX96 Touch Real-Time PCR Detection System (Bio-Rad). Data were analyzed by the delta delta Ct’ (ΔΔCt) method, and *Rab8b* expression was quantified relative to *GAPDH*.

### ChIP Assay

ChIP assays for CQ-induced or vehicle-treated basal levels of p53 binding to the Rab8b promoter or to Par-4 gene are described in the Supplemental Experimental Procedures.

### Co-localization of Par-4 in Rab8b-Coated Vesicles

Cells in chamber slides were treated with CQ or vehicle in the presence or absence of BFA and subjected to ICC analysis with Par-4 (C-19 goat) primary antibody and donkey anti-goat secondary antibody conjugated to Alexa Fluor 568 (red fluorescence; A10042, Invitrogen) and with Rab8b (rabbit) primary antibody and donkey anti-rabbit secondary antibody conjugated to Alexa Fluor 488 (green fluorescence; A11055, Invitrogen). Cells were stained with DAPI to reveal their nuclei (blue fluorescence).

### Animal Experiments

All animal experiments are described in the Supplemental Experimental Procedures.

### Statistical Analysis

All experiments were performed in triplicate to verify the reproducibility of the findings. The results show a mean of at least three experiments ± SD. Statistical analyses were carried out with Statistical Analysis System software (SAS Institute), and p values were calculated using the Student’s t test.

## Supplementary Material

1

## Highlights

- Chloroquine induces the secretion of the tumor suppressor protein Par-4
- Chloroquine-induced Par-4 secretion depends on p53
- p53 directly induces Rab8b expression critical for Par-4 secretion
- Par-4 is essential for apoptosis and inhibition of tumor metastasis by chloroquine

## In Brief

Burikhanov et al. identify the anti-malarial drug chloroquine (CQ) as a robust secretagogue of tumor suppressor Par-4. CQ-inducible Par-4 secretion is dependent on p53 and Rab8b for vesicle transport. Induction of Par-4 secretion provides an attractive option for the repurposing of existing drugs for apoptosis and inhibition of tumor metastasis.
