# Supplementary material for: Chloroquine-Inducible Par-4 Secretion Is Essential for Tumor Cell Apoptosis and Inhibition of Metastasis
Source: Cell Rep. Author manuscript; Available in PMC 2017 Jan 25. (PMC5264245; doi:10.1016/j.celrep.2016.12.051)
Supplement: 1 [file NIHMS838548-supplement-1.pdf]

Cell Reports, Volume *18*

## Supplemental Information

# Chloroquine-Inducible Par-4 Secretion Is Essential for Tumor Cell Apoptosis and Inhibition of Metastasis

**Ravshan Burikhanov, Nikhil Hebbar, Sunil K. Noothi, Nidhi Shukla, James Sledziona, Nathália Araujo, Meghana Kudrimoti, Qing Jun Wang, David S. Watt, Danny R. Welch, Jodi Maranchie, Akihiro Harada, and Vivek M. Rangnekar**

**Table S1. FDA-approved compounds screened for induction of Par-4 secretion, Related to Figure 1.**

| Compound | Drug Class | Target | Structure |
|---|---|---|---|
| Flumequine | fluoroquinolone | anti-bacterial (DNA gyrase, topoisomerase IV) | 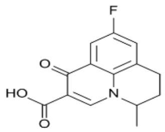 |
| Ofloxacin | fluoroquinolone | anti-bacterial (DNA gyrase, topoisomerase IV) | 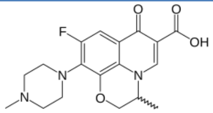 |
| Levofloxacin | fluoroquinolone | anti-bacterial (DNA gyrase, topoisomerase IV) | 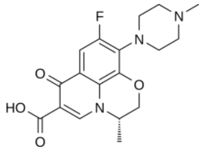 |
| Lomefloxacin hydrochloride | fluoroquinolone | anti-bacterial (DNA gyrase, topoisomerase IV) | 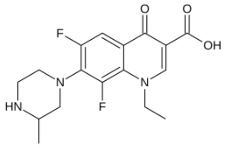 |
| Ciprofloxacin | fluoroquinolone | anti-bacterial (DNA gyrase, topoisomerase IV) | 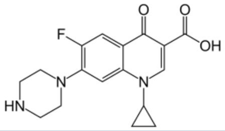 |
| Norfloxacin | fluoroquinolone | anti-bacteria cytochrome P450 | 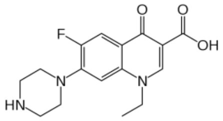 |
| Difloxacin Hydrochloride | fluoroquinolone | anti-bacterial (DNA gyrase, topoisomerase IV) | 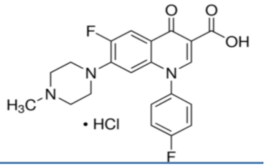 |
| Sparfloxacin | fluoroquinolone | anti-bacterial (DNA gyrase, topoisomerase IV) | 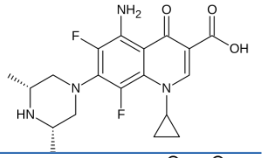 |
| Pefloxacin Mesylate | fluoroquinolone | anti-bacterial (DNA gyrase, topoisomerase IV) | 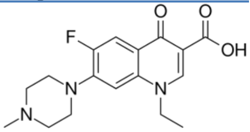 |

**Table S1 Continued**

| | | | |
|---|---|---|---|
| Enrofloxacin | fluoroquinolone | anti-bacterial (DNA gyrase, topoisomerase IV) | 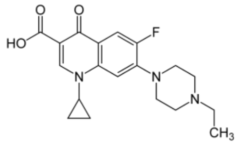 |
| Nalidixic acid | quinolone | anti-bacterial (DNA gyrase, topoisomerase IV) | 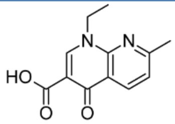 |
| Nalidixic acid sodium salt | quinolone | anti-bacterial (DNA gyrase, topoisomerase IV) | 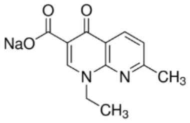 |
| Tafenoquine succinate | 8-aminoquinoline | anti-malarial | 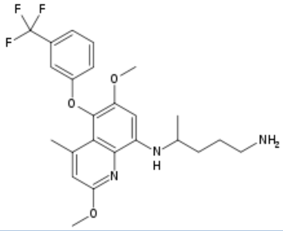 |
| Primaquine bisphosphate | 8-aminoquinoline | anti-malarial | 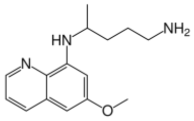 |
| Amodiaquine | 4-aminoquinoline | anti-malarial | 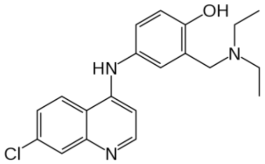 |
| Chloroquine | 4-aminoquinoline | anti-malarial/anti-autophagic | 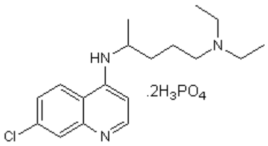 |
| Hydroxy-Chloroquine | 4-aminoquinoline | anti-malarial/anti-autophagic | 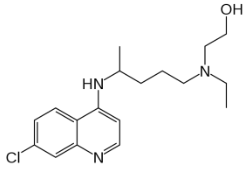 |

**Figure S1**

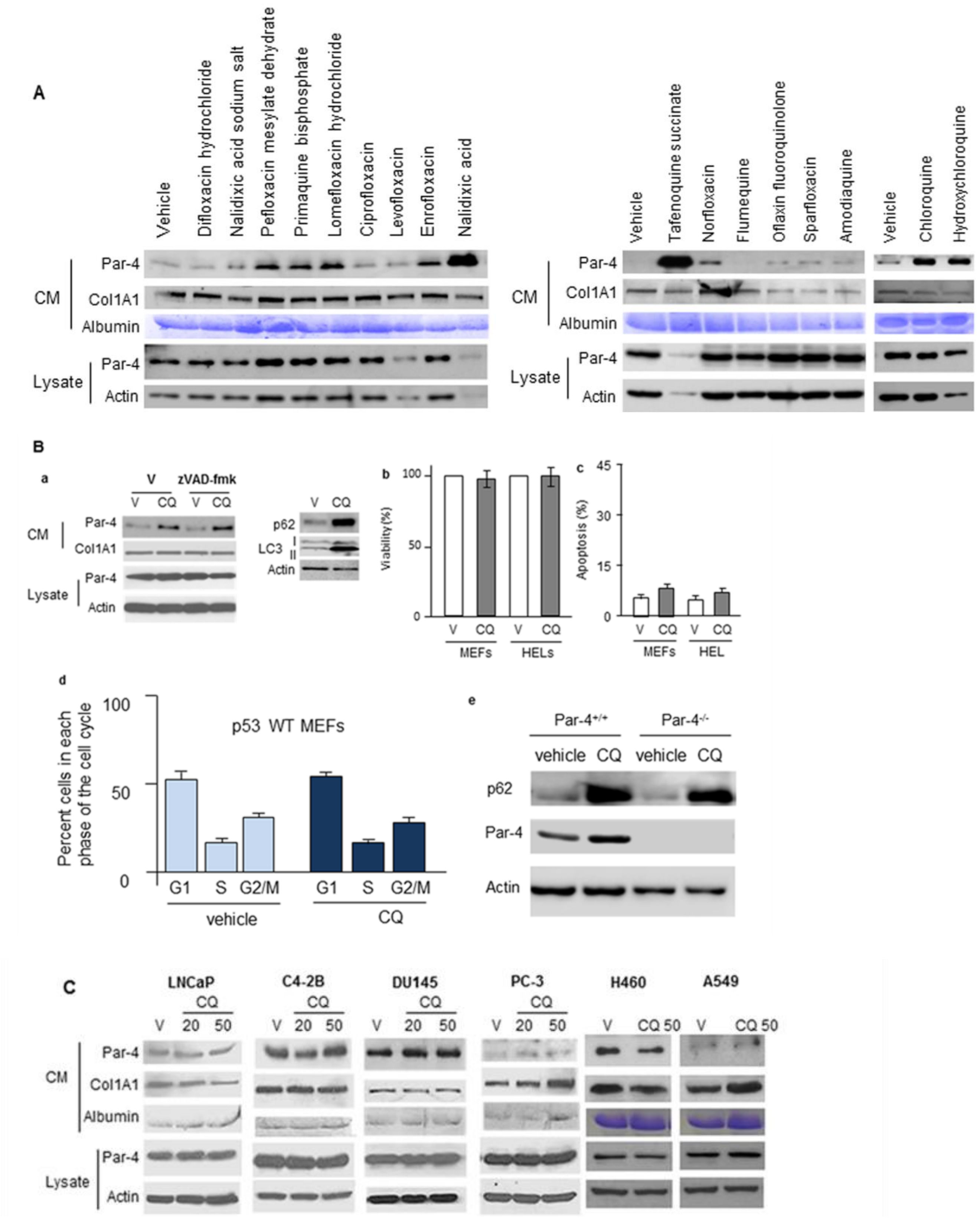
A
Vehicle
Difloxacin hydrochloride
Nalidixic acid sodium salt
Pefloxacin mesylate dehydrate
Primaquine bisphosphate
Lomefloxacin hydrochloride
Ciprofloxacin
Levofloxacin
Enrofloxacin
Nalidixic acid
Tafenoquine succinate
Norfloxacin
Flumequine
Oflaxin fluoroquinolone
Sparfloxacin
Amodiaquine
Chloroquine
Hydroxychloroquine
CM
Lysate
Par-4
Col1A1
Albumin
Actin
B
a
V
zVAD-fmk
CQ
p62
LC3
I
II
b
Viability (%)
MEFs
HELs
c
Apoptosis (%)
HEL
d
p53 WT MEFs
Percent cells in each phase of the cell cycle
G1
S
G2/M
vehicle
e
Par-4+/+
Par-4-/-
C
LNCaP
C4-2B
DU145
PC-3
H460
A549
20
50
CQ 50

**Figure S1. CQ induces Par-4 secretion from normal cells, but not cancer cells, by an apoptosis-independent mechanism, Related to Figure 1.**

**A. Screen for Par-4 secretagogues.** MEFs were treated with 25 μM amounts of generic compounds or vehicle (V) control for 24 h. The CM and whole-cell lysates were subjected to Western blot analysis with the indicated antibodies. Collagen1A1 (Col1A1) was used as a loading control for protein secretion, as it was generally unchanged in response to the treatments. The samples were also subjected to SDS-PAGE and Coomassie blue staining for albumin levels in serum from the CM, which was another loading control. Actin served as a loading control for the lysates.

**B. CQ induced Par-4 secretion by an apoptosis-independent mechanism and Par-4 did not regulate expression of the autophagy associated protein p62/SQSTM1.** HEL cells were pre-treated with zVAD-fmk (2 μM) or Vehicle (V) for 30 minutes, and in the presence of zVAD-fmk, further treated with CQ (20 μM) or V or directly treated for 24 h with V or CQ in the absence of any pretreatment. The CM and lysates were analyzed on Western blots (**a**). MEF cells or HEL cells were treated with CQ (25 μM) or V for 24 h. Percent viability in the cultures was determined by MTS Cell Proliferation Colorimetric Assay (**b**) and percent apoptosis was determined by ICC for active caspase 3 and DAPI staining (**c**). Wild type MEFs ($p53^{+/+}$) were treated with CQ (25 μM) or vehicle for 24 h, stained with propidium iodide, and cell cycle distribution was analyzed in the Becton-Dickinson LSRII flow cytometer at the Markey Cancer Center Flow Cytometry shared resource facility **(d).** Par-$4^{+/+}$ and Par-$4^{-/-}$ MEFs were treated with vehicle or CQ (20 μM) for 24 h, and whole cell extracts were examined for p62 expression by Western blot analysis **(e)**.

**C. CQ did not induce Par-4 secretion in cancer cells.** Various cancer cells were treated with CQ (20 or 50 μM) or V for 24 h and the CM and lysates were analyzed on Western blots with the indicated antibodies.

**Figure S2**

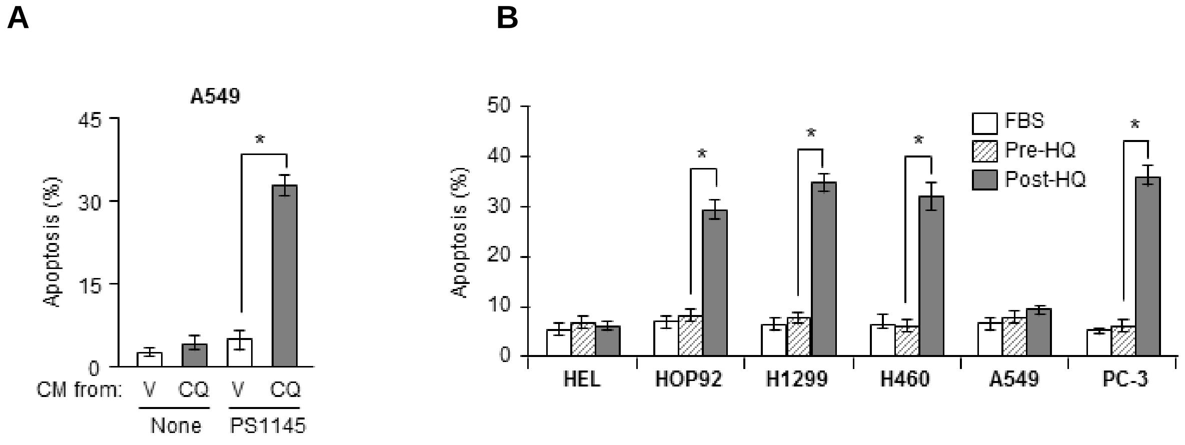


**Figure S2. Par-4 secreted in response to CQ induces apoptosis in cancer cells, Related to Figure 2.**

**A. PS-1145 sensitized Par-4 resistant A549 cells to apoptosis by Par-4 in CM from CQ treated fibroblasts**. Aliquots of CM from wild type MEFs treated with vehicle (v) or CQ were incubated with A549 cells that were pretreated with PS-1145 (10 μM) or vehicle (v). After 24 h, the cells were scored for apoptosis. * $P$<0.0001 by the Student's t-test.

**B. Plasma from CQ treated patients induces *ex vivo* apoptosis of cancer cells**. Aliquots of pre-CQ or post-CQ treatment plasma samples (20% final concentration) from patient RCC4 were transferred to cancer cell cultures or normal cells. Apoptotic cells were scored after 24 h. FBS, fetal bovine serum, was used as another control. * $P$<0.0001 by the Student's t-test.

**Figure S3**

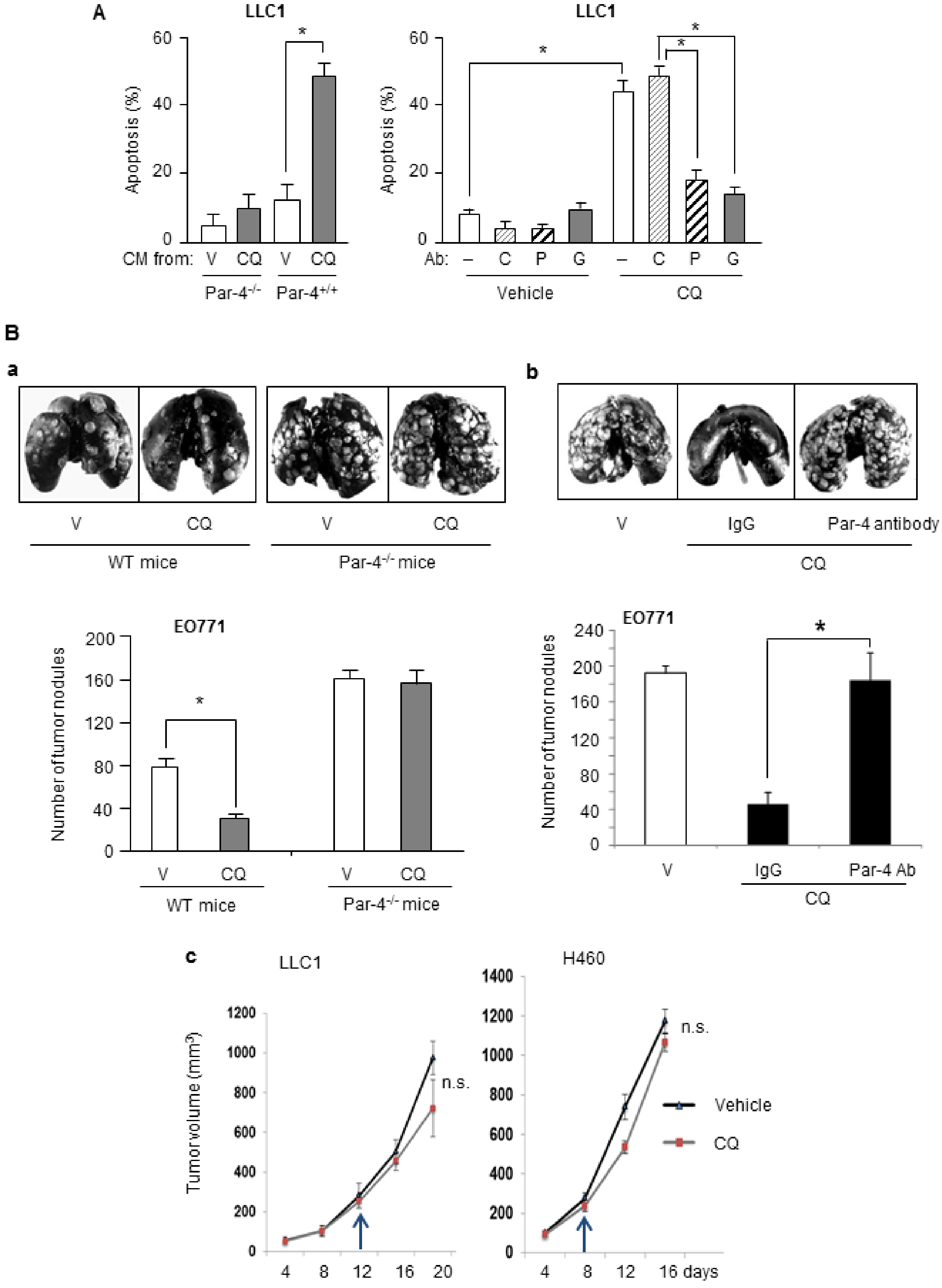
A
LLC1
Apoptosis (%)
CM from: V CQ V CQ
Par-4-/- Par-4+/+
LLC1
Ab: – C P G – C P G
Vehicle
CQ
B
a
V CQ
WT mice
V CQ
Par-4-/- mice
b
V IgG Par-4 antibody
CQ
EO771
Number of tumor nodules
V CQ
WT mice
V CQ
Par-4-/- mice
EO771
V IgG Par-4 Ab
CQ
c
LLC1
Tumor volume (mm3)
n.s.
H460
n.s.
Vehicle
CQ
4 8 12 16 days

**Figure S3. Secreted Par-4 induces cancer cell apoptosis and inhibits metastasis in a Par-4-dependent manner, Related to Figure 3.**

**A. Par-4 in CM from CQ treated MEFs induced apoptosis in LLC1 cells**. Aliquots of CM from Par-4$^{+/+}$ or Par-4$^{-/-}$ MEFs treated for 24 h with CQ (20 μM) or vehicle (v) were incubated with LLC1 cells (left panel). Moreover, aliquots of CM from wild type MEFs treated with CQ or vehicle were incubated with control (C) antibody (Ab), Par-4 (P) Ab or GRP78 (G) Ab and then transferred to LLC1 cells (right panel). After 24 h, the cells were scored for apoptosis (left and right panels). * $P$<0.0001 by the Student's t-test.

**B. CQ inhibited experimental lung metastasis in a Par-4-dependent manner, but did not inhibit the growth of subcutaneous bulky tumors.**

**a. CQ inhibits experimental metastasis of EO771 cells.** C57BL/6 Par-4$^{+/+}$ (wild type, WT) mice or Par-4$^{-/-}$ mice were injected i.v. with EO771 breast cancer cells (Tian *et al*., 2002; Methany-Barlow *et al*., 2004; Johnstone *et al*., 2015, Supplemental References) (0.5 x $10^6$ cells), and 24 h later injected i.p. with CQ (25 mg/kg body weight) or vehicle (V) once every day for 5 consecutive days. After 21 days the lungs were perfused, stained with India ink (upper panel) and tumor nodules were scored (lower panel). * $P$ < 0.001 by the Student's t-test.

**b. CQ induced Par-4 secretion is essential for inhibition of metastasis by CQ in the EO771 experimental metastasis model**. Athymic (*nu*/*nu*) mice were injected i.v. with EO771 cells (0.5 x $10^6$ cells), and 24 h later injected i.p. with CQ (25 mg/kg body weight) or vehicle (V) once every day for 5 consecutive days. Animals injected with CQ were also injected with either the control IgG or Par-4 polyclonal antibody (20 μg/injection). After 21 days, the lungs were perfused, stained with India ink (upper panel) and the tumor nodules were scored (lower panel). * $P$ < 0.001 by the Student's t-test.

**c. CQ did not inhibit the growth of subcutaneous bulky tumors.** We used 4 x$10^5$ H460 or LLC1 cells to generate subcutaneous tumors in the flanks of immunocompromised (*nu/nu*) mice. When the tumors had grown to about 200 $mm^3$ volume (indicated by arrow), the mice were injected with CQ (25 mg/kg body weight) or vehicle once daily throughout the experiment (10 mice per group). Tumor growth was followed over period of the experiment, and average tumor volumes $\pm$ SD in each cohort are shown. The difference in tumor volume between the vehicle and CQ treated groups was not significant (n.s., $P$ > 0.05) by the Student's t test.

## Figure S4

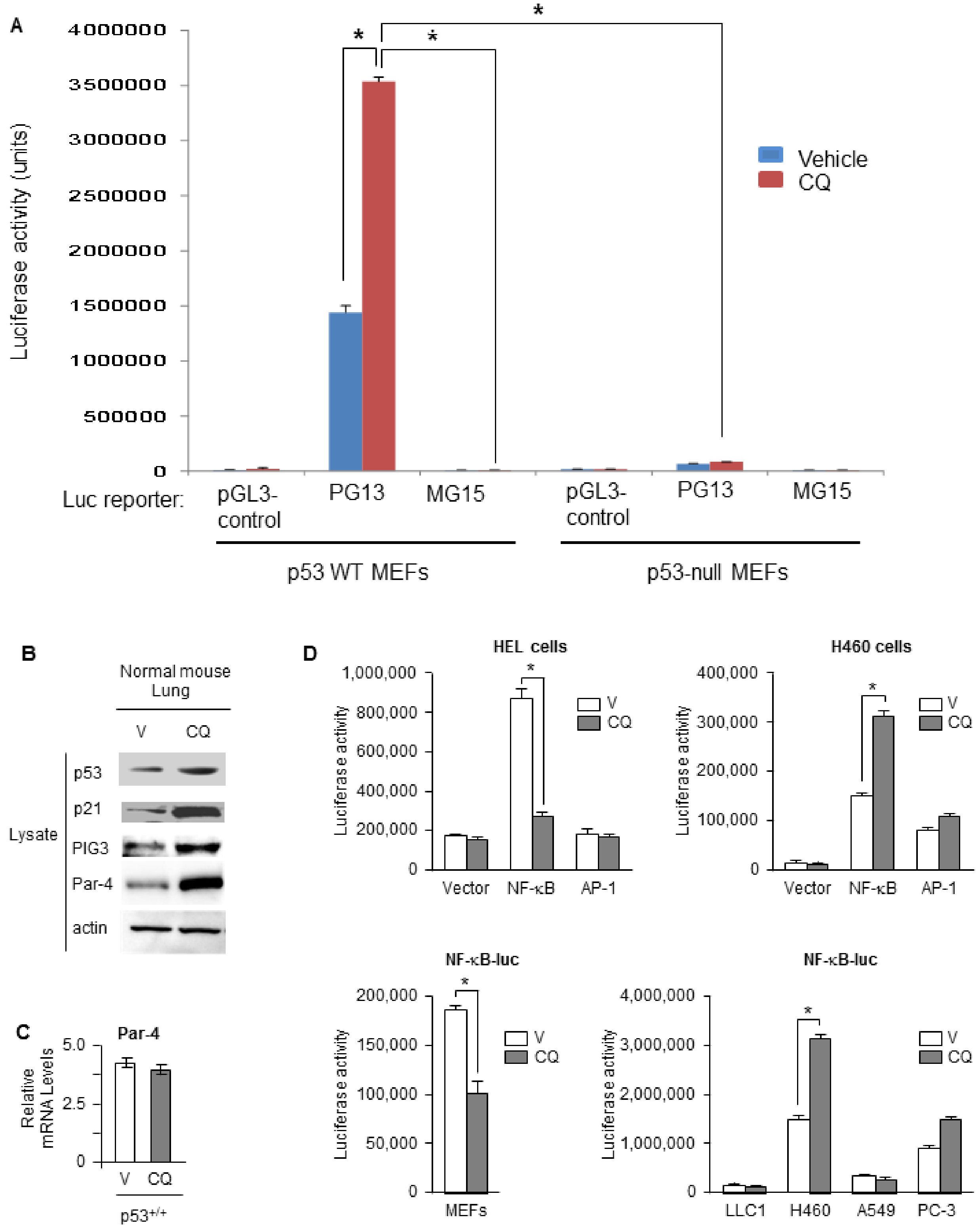

A
Luciferase activity (units)
4000000
3500000
3000000
2500000
2000000
1500000
1000000
500000
0
Vehicle
CQ
Luc reporter:
pGL3-control
PG13
MG15
pGL3-control
PG13
MG15
p53 WT MEFs
p53-null MEFs
B
Normal mouse Lung
V
CQ
Lysate
p53
p21
PIG3
Par-4
actin
C
Par-4
Relative mRNA Levels
5.0
2.5
0
V
CQ
p53+/+
D
HEL cells
Luciferase activity
1,000,000
800,000
600,000
400,000
200,000
0
V
CQ
Vector
NF-κB
AP-1
H460 cells
400,000
300,000
200,000
100,000
NF-κB-luc
200,000
150,000
100,000
50,000
MEFs
NF-κB-luc
4,000,000
3,000,000
2,000,000
1,000,000
LLC1
H460
A549
PC-3

**Figure S4. CQ induces p53 activation and inhibition of NF-κB activity, Related to Figure 4.**

**A. CQ induced p53-dependent transcription and upregulation of p53-responsive genes.** MEFs ($p53^{+/+}$ or $p53^{-/-}$) were transiently co-transfected with p53-reporter (PG13-luc containing p53 binding sites), mutant reporter (MG15-luc containing mutated binding sites for p53) or pGL3 control luc construct, and β-galactosidase expression construct. The transfectants were treated with CQ (25 μM) or vehicle for 24 h, luciferase activity was determined and normalized to corresponding β-galactosidase activity and expressed as relative luciferase activity units. CQ induced luciferase activity from the p53-reporter luciferase construct but not from a luciferase construct containing mutant response element in $p53^{+/+}$ MEFs. By contrast, CQ failed to induce luciferase activity in $p53^{-/-}$ MEFs.

**B. CQ induced upregulation of p53-responsive genes.** C57BL/6 mice were injected once daily with CQ (25 mg/kg body weight) or vehicle for five consecutive days. Blood samples were collected from the mice on the sixth day, and plasma was examined for expression of p53, p21, PIG3, Par-4, and actin by Western blot analysis.

**C. CQ did not elevate Par-4 RNA levels.** MEFs were treated with CQ (25 μM) or vehicle (V) for 24 h and mRNA prepared from the cells was examined by Real-Time quantitative reverse transcription PCR (qRT-PCR) for Par-4. The data were normalized relative to a GAPDH control.

**D. CQ inhibited NF-κB activity, but not AP1 activity in normal cells.** Normal (HEL and MEF) or cancer (LLC1, H460, A549, PC-3) cells were co-transfected with luciferase (luc) reporter construct for NF-κB, AP1, or empty luc construct and β-galactosidase expression construct and treated for 18 h with CQ (25 μM) or vehicle (V). Luciferase activity was analyzed in the cell lysates and normalized with respect to the corresponding β-galactosidase activity.

**A and D.** * $P < 0.001$ by the Student's t-test.

**Figure S5**

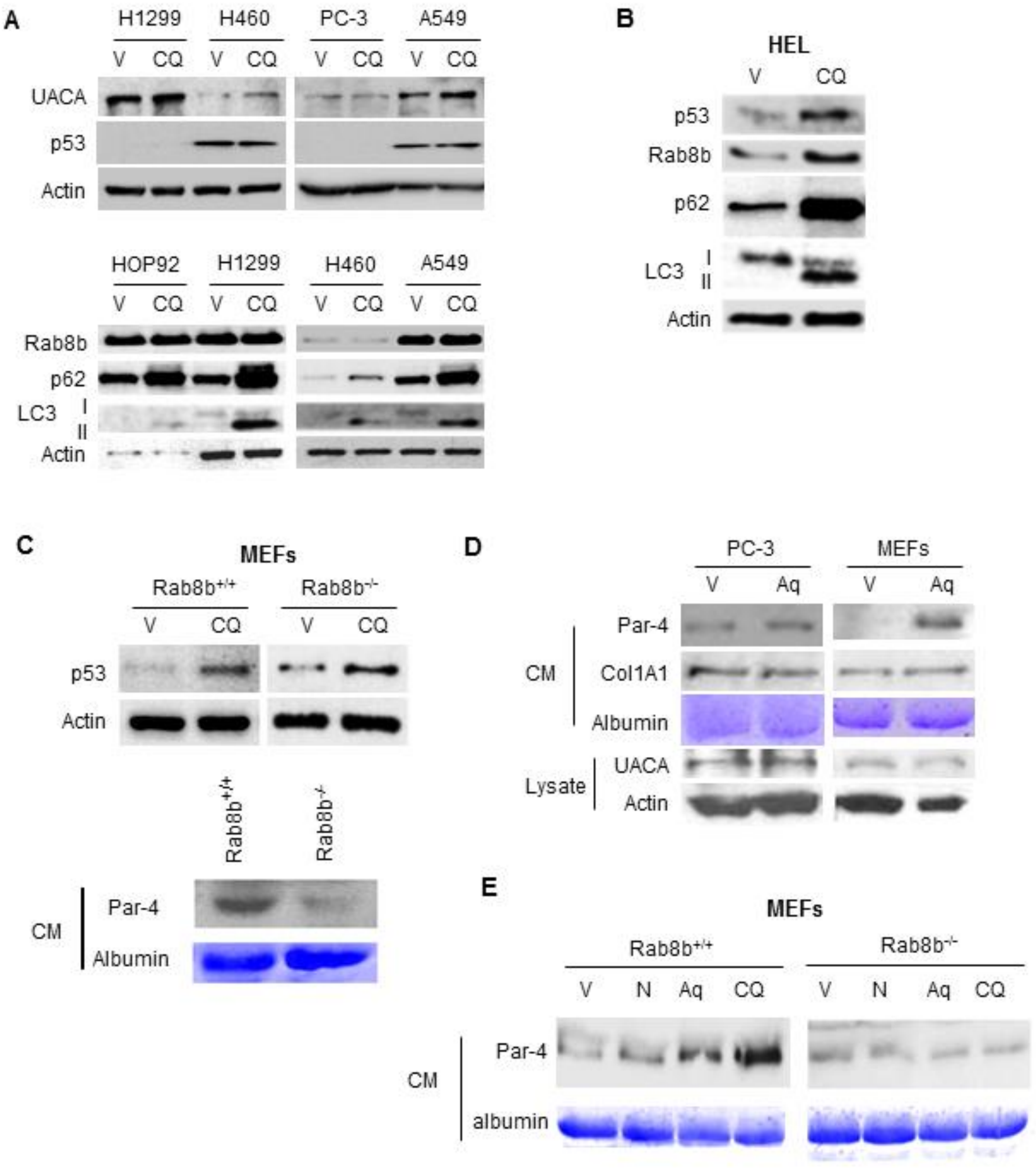


**Figure S5. CQ induces p53 and Rab8b in normal human cells but not cancer cells, Related to Figure 5.**

**A. CQ did not induce p53 or Rab8b, or UACA downregulation in cancer cells.** Various lung cancer or prostate cancer cells were treated with CQ (25 μM) or vehicle (V) for 24 h and the lysates was examined for UACA and p53 (upper panel) or Rab8b, p62 and LC3 (lower panel) by Western blot analysis.

**B. CQ induced p53 and Rab8b in normal human cells**. HEL cells were treated with CQ (25 μM) or vehicle (V) for 24 h and the lysates was examined by Western blot analysis with the indicated antibodies.

**C. CQ induced p53 expression is not compromised in Rab8b-null cells (Upper Panel).** Rab8b$^{+/+}$ or Rab8b$^{-/-}$ cells were treated with CQ (25 μM) or vehicle (V) for 24 h and the lysates were examined for p53 expression by Western blot analysis. **Rab8b is essential for optimal secretion of Par-4 (Lower Panel).** CM from Rab8b wild type and Rab8b$^{-/-}$ cells were examined for Par-4 expression by Western blot analysis.

**D. Arylquin-1, which binds to vimentin to allow Par-4 secretion (Burikhanov *et al*., 2014b), did not inhibit UACA expression**. MEFs or PC-3 cells were treated with Arylquin 1 (Aq, 500 nM) or vehicle (V) for 24 h, and the CM and lysates were examined by Western blot analysis.

**E. Nutlin-3a (N), Arylquin-1 (Aq) or CQ induced Par-4 secretion by a Rab8b-dependent mechanism**. Rab8b$^{+/+}$ or Rab8b$^{-/-}$ MEFs were treated with N (10 μM), Aq (500 nM), CQ (25 μM) or vehicle (V) for 24 h, and the CM were examined by Western blot analysis.

**Figure S6**

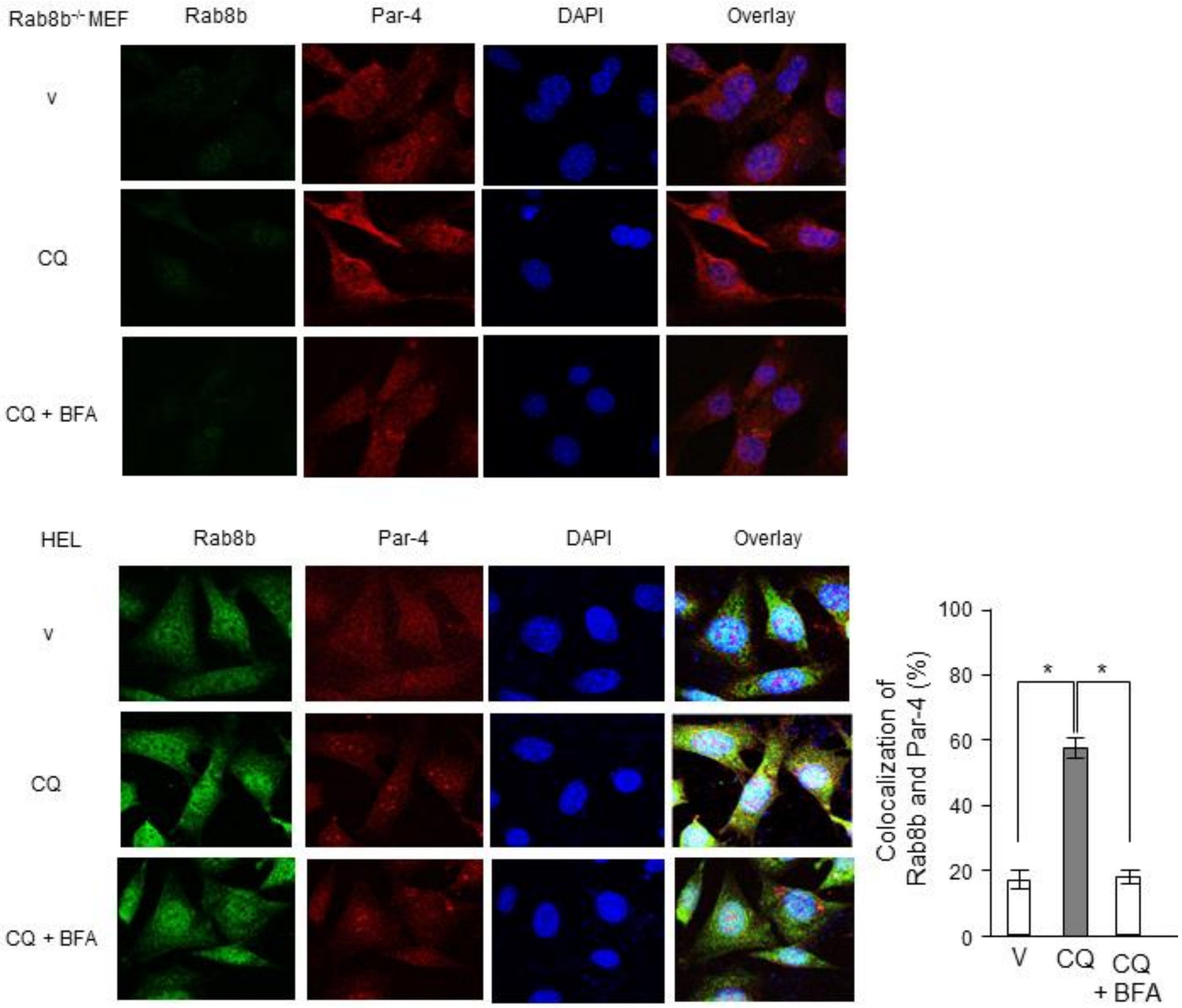


**Figure S6. Par-4 co-localizes with Rab8b+ vesicles in CQ treated cells, Related to Figure 6.** HEL cells or Rab8b$^{-/-}$ MEF cells, which were used as a control for Rab8b$^{+/+}$ MEF cells shown in Figure 6C, were treated with vehicle (V) or CQ (25 μM) in the absence or presence of BFA (1 μg/ml) for 24 h, and subjected to ICC for Par-4 (red fluorescence) and Rab8b (green fluorescence). Cells were stained with DAPI to reveal their nuclei (cyan fluorescence). The percentage of cells showing colocalization of Par-4 and Rab8b vesicles was quantified (lower right panel). In HEL cells, note dissociation of Par-4 and Rab8b (loss of yellow fluorescence, but retention of red and green fluorescence) in the CQ + BFA panel. As expected, Rab8b$^{-/-}$ MEF cells showed absence of Rab8b staining with the Rab8b antibody. * $P < 0.001$ by the Student's t-test.

## Supplemental Methods

### *Primers used for Real-Time quantitative reverse transcription PCR (qRT-PCR) assay*

The primers used were: mRab8a Forward: 5'-CTGGCACTCGACTATGGGAT-3'; mRab8a Reverse: 5'-TTTGCTTTGATATCCCTGGC-3'; mRab8b Forward: 5'-CACGCCTCTTCAGATGTTGA-3'; mRab8b Reverse: 5'-CGACTTTGCACTTGTCTCCA-3'; mouse Par-4 Forward: 5'- AGAATGAAGCTGCGACCCTC-3'; mouse Par-4 Reverse: 5'-ATCTTCTGGGGC ACTGGTTG-3'; and internal control primers for mGAPDH Forward: 5'-GTGAGGCAAAAGGGAAGGTG-3'; mGAPDH Reverse: 5'-AGGCATGGCAGATTCAG AGT-3'.

### *Chromatin-Immunoprecipitation (ChIP) Assay*

ChIP assays for CQ-induced or vehicle-treated basal levels of p53 binding to the Rab8b promoter or to Par-4 gene involved treatment of wild type MEFs (2 x$10^6$ cells) with vehicle or CQ (25 uM) for 8-12 Hrs. ChIP analysis was performed by using the ChIP Assay kit from Millipore or Active Motif according to the instructions provided by the manufacturer. Sheared chromatin was immunoprecipitated with antibodies using mouse IgG (sc-2025, Santa Cruz Biotechnology), or anti-p53 antibody (1C12 mouse monoclonal antibody #2524 from Cell Signaling. DNA fragments were amplified by using validated primers for p53 binding site on the Rab8b promoter from EpiTect ChIP qPCR primer assay for mouse Rab8b (GPM1054933(-)01A; Qiagen) and resolved on agarose gels, or quantitative PCR was performed on CFX96 Touch™ Real-Time PCR Detection System (Bio-Rad). Two different primer sets: (1) Forward 5' TCACCTGAGCAAAACGACGA 3'; Reverse 5' GCGATCAGAGCTAAGGGGAC 3'; (2) Forward 5' GGCTGAGCCTGTCCTCTTTC 3'; Reverse 5' GTGCTGTGTTGGTTTCTCGG 3'; were used along the Par-4 gene, which does not contain a p53-binding site. GAPDH control primers used were: Forward 5'-ATG GTT GCC ACT GGG GAT CT-3'; Reverse 5'-TGC CAA AGC CTA GGG GAA GA-3'.

### *Animal experiments*

To determine whether CQ induced Par-4 secretion in immunocompetent mice, C57BL/6 mice were injected via the intraperitoneal (i.p.) route with a single injection of CQ (50 mg/kg body weight) or vehicle, and whole-blood samples were collected 24 h later. Plasma was separated from the blood samples, heated at 56$^{o}$C to inactivate complement. Aliquots of the mouse plasma samples were added to the growth medium (final 20% mouse plasma) of normal and cancer cells in culture and tested for induction of *ex vivo* apoptosis in cancer cells. To test the effect of CQ on metastatic growth of tumors, EO771 cells or LLC1 cells expressing luciferase (0.5 x $10^6$ cells) were injected via the tail vein in Par-4$^{+/+}$ (wild type) or Par-4$^{-/-}$ C57BL/6 mice and 24 h later, the mice were injected i.p. with CQ (25 mg/kg body weight) injection given daily for five consecutive days. Each group included 8 mice. Plasma from the mice was examined 24 h after the 5th CQ injection for Par-4 expression. The mice were imaged for luciferase expression as previously described (Zhao *et al*., 2011), and humanely killed to examine

their tumors at day 21. The lungs were then perfused, stained with India ink and the tumor nodules were scored as previously described (Zhao *et al*., 2011).

To test whether Par-4 secretion induced by CQ was involved in inhibition of LLC1- or EO771-derived metastatic tumor growth, we used an experimental metastasis model. Athymic (nu/nu) mice were injected i.v. with 500 x $10^5$ LLC1 cells, and 24 h later injected i.p. with CQ (25 mg/kg body weight) or vehicle once every day for 5 consecutive days. Animals injected with CQ were also injected within 2 h with either the control IgG or Par-4 polyclonal antibody (20 μg/injection). Each group included 10 mice. After 21 days, the lungs were perfused, stained with India ink and the tumor nodules were scored.

To determine whether CQ inhibits the growth of large bulky tumors, we injected 4 x $10^5$ H460 or LLC1 cells subcutaneously in nude (*nu/nu*) mice to generate tumors in the flanks. When the tumors had grown to *ca*. 200 $mm^3$ volume (indicated by arrow), the mice (10 per group) were injected with CQ (25 mg/kg body weight) or vehicle once daily throughout the experiment, and tumor growth was measured with calipers over period of the experiment to calculate tumor volume. All animal procedures were performed with University of Kentucky IACUC approval.

## *Supplemental References*

Johnstone CN, Smith YE, Cao Y, Burrows AD, Cross RSN, Ling X, *et al*. (2015) Functional and molecular characterisation of EO771.LMB tumours, a new C57BL/6-mouse-derived model of spontaneously metastatic mammary cancer. Dis Model Mech 8: 237–251.

Metheny-Barlow LJ, Tian S, Hayes AJ, and Li LY. (2004) Direct chemotactic action of angiopoietin-1 on mesenchymal cells in the presence of VEGF. Microvasc.Res. 68:221-230.

Tian S, Hayes AJ, Metheny-Barlow LJ, and Li LY. (2002) Stabilization of breast cancer xenograft tumour neovasculature by angiopoietin-1. Br.J.Cancer 86:645-651.
